# STEPS (Study To Examine Parent, Patient/Dental Provider Systems) to Prevent Human Papillomavirus (HPV)-Related Cancers: A Piloted Dental Patient and Provider Evaluation of Current and Future HPV Education

**DOI:** 10.1007/s13187-024-02465-2

**Published:** 2024-07-04

**Authors:** Kelsey H. Jordan, Julie A. Stephens, Kaleigh Niles, Nina Hoffmeyer, Michael L. Pennell, Jill M. Oliveri, Electra D. Paskett

**Affiliations:** 1 Division of Population Sciences, Comprehensive Cancer Center, The Ohio State University, Columbus, OH USA; 2 Center for Biostatistics, Department of Biomedical Informatics, College of Medicine, The Ohio State University, Columbus, OH USA; 3 Recruitment, Intervention and Survey Shared Resource, Comprehensive Cancer Center, The Ohio State University, Columbus, OH USA; 4 Division of Biostatistics, College of Public Health, The Ohio State University, Columbus, OH USA; 5 Division of Epidemiology, College of Public Health, The Ohio State University, Columbus, OH USA; 6 Division of Cancer Prevention and Control, Department of Internal Medicine, College of Medicine, The Ohio State University, Columbus, OH USA

**Keywords:** Human papillomavirus (HPV), Head and neck neoplasms, Oropharyngeal cancer, Preventive dentistry, (Cancer or papillomavirus) vaccine, Patient education handout, (Continuing) dental education

## Abstract

Oropharyngeal human papillomavirus (HPV) cancers are prevalent, but HPV education in dental clinics is uncommon. The purpose of this study was to evaluate dental provider and patient knowledge from, attitudes towards, and preferences for HPV education, then assess perceptions of existing HPV educational materials for use at dental visits. Appalachian Ohio dental patients (*n* = 13) and general/pediatric dental providers (*n* = 10) completed an initial, close-ended survey on current HPV knowledge and HPV educational attitudes, participation, and resource preferences. Select individuals reviewed existing HPV educational videos and toolkits via virtual focus groups (*n* = 9) or independent review surveys (*n* = 6). Using a discussion guide, participants responded to overall, visual, auditory, and content satisfaction statements, orally (focus groups) or with Likert scales (independent reviews). Surveys were summarized with frequencies/percentages; transcripts were qualitatively coded to identify potential material modifications. Dental providers and patients were more comfortable with HPV and oral cancer education (87% and 96%, respectively) and screening (96%) than with HPV vaccine education (74%) and referrals (61%) during dental visits. Providers were neither sharing HPV educational materials (80%) nor initiating educational conversations with dental patients (100%). The American Cancer Society videos and the “Team Maureen” toolkit were the most liked resources (i.e., fewer negative/disagree statements) by all participant groups. Findings indicate that future dental HPV educational efforts should be informed by currently available materials. Additional interventions are needed to promote dental provider discussions and sharing of educational materials with patients to increase education and promotion of the HPV vaccine and reduce oropharyngeal cancers.

**Supplementary Information:**

The online version contains supplementary material available at 10.1007/s13187-024-02465-2.

## Introduction

Three in four United States (US) oropharyngeal cancer diagnoses are linked to human papillomavirus (HPV) [1]. In medically underserved areas like Appalachia, HPV cancer rates exceed national data. The 2019 age-adjusted oral cancer incidence rate (per 100,000 people) was 7.1 in Appalachia and 5.2 nationally [2, 3]. The Gardasil-9 vaccine can protect against these cancers [4], but US vaccination rates (goal, 80%) remain sub-optimal, especially in 13–17 year olds (USA: 59%; Appalachia: 43%). Limited parent HPV vaccine knowledge and lack of a strong provider recommendation continue to be the main reasons children remained unvaccinated nationally [5, 6]. Vaccination rates are even lower in adults, 27–45 year olds, who must first discuss it with their provider [6–8].

National dental professional groups encourage dentists to educate patients about HPV and make strong recommendations for HPV vaccination, without mentioning specific patient educational materials to share [9, 10]. Dental providers desire written, plain language educational materials such as posters, brochures, question and answer sheets, and brief videos to share with patients [11–13]. Dentist and hygienist specific request for American Dental Association, ADA, branded educational commercials and pamphlets further reiterates the current professional support deficiency [12].

Other organizations (e.g., American Cancer Society, Centers for Disease Control, Team Maureen) have created various HPV educational materials freely available to the public with limited (e.g., New England area, USA only) or no provider and/or patient evaluation. Providers must locate and evaluate these materials independently [14–17]. Despite these resources, providers dedicate negligible amounts of time, if any, informing patients about HPV [11, 15, 18–22]. Parents prefer written educational materials, but 90% of surveyed parents have not received the HPV educational information they wanted during dental visits. Additional patient education preferences remain unknown as these studies are rare. When investigated, prior studies have not assessed HPV knowledge levels and have excluded vaccine-eligible 27–45 year old adults from participation [23, 24].

Dental providers can offer patients a critical perspective on the importance of the HPV vaccine to prevent HPV-related oral cancers. Regrettably, dental HPV educational efforts are limited, and existing HPV materials have less emphasis on oral health and men. The purpose of this study was to evaluate Appalachian dental provider and patient HPV knowledge, attitudes, and preferences about educational materials and delivery methods, and then assess perceptions of existing HPV educational materials for dental visit use.

## Methods

### Eligibility Criteria

#### Recruitment Area

Study participation was restricted to English-speaking dental patients and providers residing in Appalachian Ohio. To obtain a diverse sample within Appalachian Ohio, participants were purposefully recruited from rural and urban counties, identified by Rural-Urban Continuum Codes (RUCC) scores (scores 1–3: metro; 4–9: nonmetro) [25]. Recruitment efforts were initially concentrated in Washington (rural) and Mahoning (urban) counties and expanded to surrounding counties (i.e., Trumbull, Columbiana, Athens, Morgan, and Noble) due to slower than expected recruitment during the COVID-19 pandemic. Appalachian Ohio, like all Appalachia, is a predominantly white, sociodemographically disadvantaged US region with low education and socioeconomic and health statuses [2]. The Ohio State University Institutional Review Board approved all study protocols.

#### Dental Patients

All dental patients, including parents of young dental patients and adult dental patients, were recruited for 3 months through community (zip code)-targeted dental clinic fliers (with phone screening) and Facebook advertisements (with an interest survey), each collecting contact details. Dental patients must have received general or pediatric dental care in Appalachian Ohio within the last 3 years and verbally provided informed consent. Parents/legal guardians of young dental patients had to have at least one son or daughter ages 9–17 years old; adult dental patients had to be 18–45 years old. Study-trained staff made at least three call and three email attempts each to confirm eligibility.

#### Dental Providers

All general and pediatric dentists (*n* = 241) and dental hygienists (*n* = 174) with an Ohio State Dental Board-provided mailing address within an Appalachian Ohio county of interest were emailed a survey invitation and link. All emailed providers were doubly verified by research staff to provide dental care in study counties of interest. Providers received bi-weekly reminder emails and alternating bi-weekly phone calls for 3 months. Eligible providers confirmed they offered dental care in an Appalachian Ohio county of interest and provided informed consent by voluntarily returning the completed survey.

### Surveys

#### Personal Characteristics Surveys

All participants provided basic demographic data, self-reported HPV history, and dental clinic characteristics. Parents answered questions both about themselves and their children. Dental providers also quantified their professional work history.

Most questions were multiple choice (initial: 10; material review: 13–14 questions), and a few were free response (initial: 1; material review: ≤ 5 free responses). Demographic-related data were collected at each survey phase of the study and length depended on participant group (initial: 11; material review: 12–19 questions). Study staff made bi-weekly call and email reminders for at least 2 months for any incomplete surveys throughout all study phases.

#### Baseline Surveys on Current and Preferred HPV Educational Information

Baseline surveys (see Online Resource 1) ranged in length by participant group and included questions about HPV and HPV vaccine knowledge and attitudes, current dental visit experiences with HPV education and oral cancer screenings, and future HPV educational preferences. Responses included true/false (providers: 2; parents: 3; adults: 3 questions), multiple-choice (providers: 21; parents: 19; adults: 22 questions), and 5-point Likert scales (providers: 30; parents: 15; adults: 13 questions). For some multiple-choice questions, respondents could select more than one response. Respondents completed the survey electronically or on paper with pre-paid postage provided. They received a $10 gift card for survey completion.

### HPV Educational Material Review

Select baseline surveyed patients and providers opted-in for part two of the study to review existing HPV educational videos and toolkits for dental visit use (see Online Resource 2). Participants provided feedback via a focus group or independent review survey. All participants verbally consented and received a $25 gift card for participation.

All participants watched four local/national group, institutional, and governmental videos. Two community-specific toolkits each containing posters, pamphlets, fact sheets, vaccine reminders, and/or talking tips were reviewed by all participants. Dental providers also evaluated three additional provider training national group and governmental videos and an additional medical organization-sponsored poster. Descriptions and links to all resource materials reviewed within this study can be found in the additional resources associated with this manuscript (see Online Resource 3). For videos, participants provided detailed overall and explicit audio, visual, and content feedback. For toolkits, overall appearance and content were assessed.

#### Focus Groups

Focus groups were 90 min, occurred virtually (Zoom Video Communications, Inc., San Jose, CA, USA), and were patient- or provider-specific. Meetings were capped at six participants each. Specialists conducted each meeting, utilizing Krueger techniques [26]; the moderator used guides (see Online Resource 4) to facilitate discussions with clarifying and probing questions. Each video discussion guide included eleven (three overall, two audio, one visual, five content topic) questions; eleven (three overall appearance, eight content topic) questions were also used to evaluate the print materials. The same staff member who unobtrusively recorded field notes assisted each meeting to maximize internal validity. Meetings were audio and video-recorded for playback during analyses for clarification.

#### Independent Review Surveys

Participants were emailed a REDCap survey link. Open-ended focus group discussion guide questions were rewritten into statement form for the survey (see Online Resource 5). While reviewing educational materials, participants responded to their level of agreement with the statements by using a 5-point Likert scale, adding other comments as free response. Dental providers completed 134 material review questions (100 Likert scale questions, 34 free responses). Patients, both adults with and without children, evaluated the materials by answering 80 questions (60 Likert scale questions, 20 free responses).

### Analyses

For survey data, multi-level categorical variables, like Likert scales, were collapsed into fewer levels (e.g., “strongly agree” and “agree” became “agree”; remaining responses, including “prefer not to answer” and “neither agree nor disagree,” became “not agree”), depending on data distributions. Categorical levels were reported as frequencies and percentages. Continuous variables were summarized as groups of numerical ranges. Summary data were descriptively compared within and across groups.

For focus group data, two independent coders each developed a draft codebook from one focus group transcript, compared and reached consensus on initial codes to create an initial congruent codebook, independently reapplied the congruent codebook to the initial transcript, and resolved any inconsistent coding. The process was repeated until all transcripts were independently coded and reviewed for acceptable agreement values (*κ* ≥ 0.85). Analyses were performed with NVivo qualitative software (QRS International Pty. Ltd., Burlington, MA, USA). Discussion guides were used to create an initial analysis outline. The outline supplemented the coded transcripts for a comprehensive analysis of the focus group data. Common educational material type trends were to be identified from the qualitative analysis. Survey feedback, using Likert scales, were summarized as frequencies and percentages to assess material satisfaction.

Due to small sample sizes, all results were descriptive in nature. All survey responses were stored in REDCap (Research Electronic Data Capture, Nashville, TN, USA), a HIPAA compliant electronic database [27, 28]. All data management and analyses were conducted in SAS 9.4 (SAS Institute Inc., Cary, NC, USA).

## Results

### Personal Characteristics Survey (*n* = 23)

Dental providers and patients (Table 1) were similar—mostly women (*n* = 21, 91%), White (*n* = 22, 96%), non-Hispanic (*n* = 21, 91%), and married/co-habitated (*n* = 13, 68%). All providers and parents were college-educated (vs. 38% of adults (*n* = 3)).

**Table 1:** Rural and urban Appalachian Ohio dental clinician and patient demographic characteristics

	Level (referent)	Hygienists (*n*=6)	Dentists (*n*=4)	Dental Provider Totals (*n*=10)	Parents (*n*=5)	Adults (*n*=8)	Patient Totals (*n*=13)
**Demographics**							
Gender	Female (vs. male)	6 (100%)	2 (50%)	8 (80%)	5 (100%)	8 (100%)	13 (100%)
Age	36+ years old (vs. < 36)	4 (67%)	1 (25%)	5 (50%)	4 (80%)	4 (50%)	8 (62%)
Ethnicity	Non-Hispanic (vs. Hispanic)	6 (100%)	3 (75%)	9 (90%)	4 (80%)	8 (100%)	12 (92%)
Race	White	6 (100%)	4 (100%)	10 (100%)	5 (100%)	7 (88%)	12 (92%)
	Black	0 (0%)	0 (0%)	0 (0%)	0 (0%)	1 (13%)	1 (8%)
Marital Status	Married/Cohabitate (vs. not)	2 (100%)*	2 (50%)	4 (67%)**	4 (80%)	5 (63%)	9 (69%)
Rurality	Rural (vs. urban)	1 (17%)	1 (25%)	2 (20%)	3 (60%)	4 (50%)	7 (54%)
Education	College+ (vs. < college)	2 (100%)*	4 (100%)	6 (100%)**	5 (100%)	3 (38%)	8 (62%)
Job Status	Full-time (vs. not)	0 (0%)*	4 (100%)	4 (67%)**	3 (60%)	4 (50%)	7 (54%)
**Dentistry**							
Time in career	0–10 yrs.	3 (50%)	3 (75%)	6 (60%)	----------------	----------------	------------------
	11–20 yrs.	2 (33%)	0 (0%)	2 (20%)	----------------	----------------	------------------
	21+ yrs.	1 (17%)	1 (25%)	2 (20%)	----------------	----------------	------------------
Practice Type	Private (vs. not)	4 (67%)	3 (75%)	7 (70%)	5 (100%)	7 (88%)	12 (92%)
Patient Population	General (vs. pediatrics)	1 (50%)*	4 (100%)	5 (83%)**	4 (80%)	7 (88%)	11 (85%)
Insurance	Employer/private	2 (100%)*	4 (100%)	6 (100%)**	----------------	----------------	5 (56%)***
	Medicaid/Medicare	1 (50%)*	3 (75%)	4 (67%)**	----------------	----------------	4 (44%)***
**Health Status**							
HPV infection	No (vs. yes)	6 (100%)	2 (50%)	8 (80%)	4 (80%)	4 (50%)	8 (62%)
HPV vaccine	No (vs. yes)	5 (83%)	1 (25%)	6 (60%)	5 (100%)	6 (75%)	11 (85%)
**Children**	Yes (vs. no)	2 (33%)	0 (0%)	2 (20%)	5 (100%)	----------------	5 (100%)****
Age and Sex (*n*=11)							
9–10 F	Yes (vs. no)	0 (0%)	---------------	0 (0%)	0 (0%)	---------------	0 (0%)
9–10 M	Yes (vs. no)	0 (0%)	---------------	0 (0%)	1 (11%)	---------------	1 (11%)
11–13 F	Yes (vs. no)	1 (50%)	---------------	1 (50%)	4 (44%)	---------------	4 (44%)
11–13 M	Yes (vs. no)	0 (0%)	---------------	0 (0%)	1 (11%)	---------------	1 (11%)
14–17 F	Yes (vs. no)	0 (0%)	---------------	0 (0%)	3 (33%)	---------------	3 (33%)
14–17 M	Yes (vs. no)	1 (50%)	---------------	1 (50%)	0 (0%)	---------------	0 (0%)
Ethnicity (*n*=11)	Not Hispanic (vs. Hispanic)	2 (100%)	---------------	2 (100%)	9 (100%)	---------------	9 (100%)
Race (*n*=11)	White (vs. not)	2 (100%)	---------------	2 (100%)	9 (100%)	---------------	9 (100%)
HPV vaccinated (*n*=2)	No (vs. yes)	2 (100%)	---------------	2 (100%)	6 (67%)	---------------	6 (67%)

*Only 2 hygienists provided marital status; **n=6; ***n=9; ****n=5 (adults not asked)

#### Dental Providers (*n* = 10; 6 hygienists, 4 dentists)

Most (Table 1) worked in urban areas (*n* = 8, 80%), private clinics (*n* = 7, 70%), and practiced general (vs. pediatric) dentistry (*n* = 5/6, 83%) for ≤ 10 years (*n* = 6, 60%). Half the dental providers (*n* = 5), namely hygienists, were 36 years old or older. More dentists (*n* = 3, 75%) were vaccinated than hygienists (*n* = 1, 17%). No providers’ children (*n* = 2, 100%) were vaccinated.

#### Dental Patients (*n* = 13; 5 parents, 8 adults)

Many (Table 1) were rural residents (*n* = 7, 54%) and held full-time jobs (*n* = 7, 54%). Most dental patients (*n* = 8, 62%) were 36 years old or older, especially parents (*n* = 4, 80%). Most received dental care from private clinics (*n* = 12, 92%) and general dentists (*n* = 11, 85%). More adult patients were vaccinated against HPV (*n* = 2, 25%) than parents (*n* = 0, 0%). Most (*n* = 6/9, 67%) children were unvaccinated.

### Baseline Surveys on Current and Preferred HPV Educational Information

#### Attitudes and Experiences (Data Not Shown)

Most dentists (*n* = 3, 75%) and patients (*n* = 12, 92%) were knowledgeable about HPV (> 50% questions correct). Fewer hygienists were informed (*n* = 2, 33%). Collectively, most providers and patients approved of HPV cancer education (*n* = 20, 87%), oral cancer education (*n* = 22, 96%), and oral cancer screenings (*n* = 22, 96%); they were less comfortable with HPV vaccine education (*n* = 17, 74%) and referrals (*n* = 14, 61%) for patients.

##### Dental Providers

All providers (*n* = 10) said 18–45 year olds should be screened for oral cancer. All dentists (*n* = 4) also felt it appropriate to screen 9–17 year olds; proportionally, fewer hygienists felt it appropriate (*n* = 4/6, 67%). The frequency of always screening for oral cancer increased with patient age (≤ 8 year olds: *n* = 2, 20%; 9–17 year olds: *n* = 3, 30%; 18 + year olds: *n* = 13/20, 65%). Lack of materials was the main reason (*n* = 8, 80%) for not educating patients.

##### Dental Patients

Patients were less aware (*n* = 5, 38%) of oral cancer screenings occurring during visits. No patients said HPV education or vaccine information were provided during dental visits. Cancer risk was patients’ greatest HPV concern (*n* = 12, 92%); safety was their most common (*n* = 9, 69%) vaccine concern.

### HPV Educational Preferences

#### Dental Providers

Assessing potential HPV educational material topics independently (Table 2), function of vaccine in the body and vaccine schedule were two of the most frequently identified important provider topics (80% each). Most agreed and more frequently chose dentist (90%) and medical doctor (90%) among all healthcare personnel potentially deemed appropriate for educating on HPV. Hygienist was also consistently selected by hygienists; only one dentist thought HPV education should be presented by a hygienist. Providers most frequently selected brochures as the preferred educational material (90%); they did not have strong feelings on aesthetic attributes for educational content display (Table 2).

**Table 2:** Human papillomavirus (HPV) educational knowledge preferences for Appalachian Ohio general and pediatric dental providers (i.e., dentists, hygienists) and patients (i.e., parents of young (9–17 year old) patients, adult patients with no children)

Variable	Response	Hygienist (*n*=6)	Dentist (*n*=4)	Provider Totals (*n*=10)	Parent (*n*=5)	Adult (*n*=8)	Patient Totals (*n*=13)
**Preferred HPV education topics**							
**HPV/oral cancer**							
Disease descriptions	Important	6 (100%)	4 (100%)	10 (100%)	5 (100%)	7 (88%)	12 (92%)
At-risk persons	Important	5 (83%)	4 (100%)	9 (90%)	5 (100%)	7 (88%)	12 (92%)
Screening steps	Important	6 (100%)	4 (100%)	10 (100%)	5 (100%)	8 (100%)	13 (100%)
Statistics	Important	4 (67%)	4 (100%)	8 (80%)	5 (100%)	7 (88%)	12 (92%)
**HPV vaccine**							
Functionality	Important	4 (67%)	4 (100%)	8 (80%)	5 (100%)	7 (88%)	12 (92%)
Cost	Important	4 (67%)	2 (50%)	6 (60%)	2 (40%)	6 (75%)	8 (62%)
Schedule	Important	4 (67%)	4 (100%)	8 (80%)	5 (100%)	7 (88%)	12 (92%)
Ages given	Important	4 (67%)	3 (75%)	7 (70%)	5 (100%)	7 (88%)	12 (92%)
Talking points	Important	4 (67%)	3 (75%)	7 (70%)	------------	------------	------------
Misperceptions	Important	1 (17%)	0 (0%)	1 (10%)	1 (20%)	0 (0%)	1 (8%)
**Preferred material educator**							
Public health staff	Yes	4 (67%)	4 (100%)	8 (80%)	2 (40%)	4 (50%)	6 (46%)
Medical doctor	Yes	6 (100%)	3 (75%)	9 (90%)	4 (80%)	7 (88%)	11 (85%)
Nurse	Yes	4 (67%)	1 (25%)	5 (50%)	2 (40%)	5 (63%)	7 (54%)
Dentist	Yes	6 (100%)	3 (75%)	9 (90%)	4 (80%)	7 (88%)	11 (85%)
Hygienist	Yes	6 (100%)	1 (25%)	7 (70%)	1 (20%)	6 (75%)	7 (54%)
Dental assistant	Yes	3 (50%)	1 (25%)	4 (40%)	0 (0%)	6 (75%)	6 (46%)
Oral cancer patient	Yes	5 (83%)	3 (75%)	8 (80%)	1 (20%)	3 (38%)	4 (31%)
**Preferred material format**							
Presentations	Yes	2 (33%)	1 (25%)	3 (30%)	------------	------------	------------
Posters	Yes	3 (50%)	1 (25%)	4 (40%)	5 (100%)	7 (88%)	12 (92%)
Brochures	Yes	5 (83%)	4 (100%)	9 (90%)	2 (40%)	5 (63%)	7 (54%)
Fact sheets	Yes	4 (67%)	2 (50%)	6 (60%)	4 (80%)	6 (75%)	10 (77%)
Talking tips	Yes	3 (50%)	1 (25%)	4 (40%)	1 (20%)	6 (75%)	7 (54%)
Newsletters	Yes	3 (50%)	1 (25%)	4 (40%)	1 (20%)	3 (38%)	4 (31%)
Additional resource lists	Yes	2 (33%)	1 (25%)	3 (30%)	1 (20%)	5 (63%)	6 (46%)
Videos	Yes	2 (33%)	1 (25%)	3 (30%)	1 (20%)	3 (38%)	4 (31%)
**Preferred statistics presentation**							
Stories	Yes	3 (50%)	2 (50%)	5 (50%)	4 (80%)	7 (88%)	11 (85%)
Bar charts	Yes	5 (83%)	4 (100%)	9 (90%)	3 (60%)	4 (50%)	7 (54%)
Numbers/percentages	Yes	6 (100%)	4 (100%)	10 (100%)	5 (100%)	8 (100%)	13 (100%)
**Preferred material colors**							
	None	6 (100%)	4 (100%)	10 (100%)	4 (80%)	6 (75%)	10 (77%)
	Inc. colors	0 (0%)	0 (0%)	0 (0%)	1 (20%)	2 (25%)	3 (23%)
	Exc. colors	0 (0%)	0 (0%)	0 (0%)	1 (20%)	2 (25%)	3 (23%)
**Preferred text**							
	None	5 (83%)	4 (100%)	9 (90%)	0 (0%)	3 (38%)	3 (23%)
	Bullet pts.	1 (17%)	0 (0%)	1 (10%)	5 (100%)	5 (63%)	10 (77%)
**Preferred pictures**							
	None	5 (83%)	4 (100%)	9 (90%)	1(20%)	2 (25%)	3 (23%)
	Real pictures	1 (17%)	0 (0%)	1 (10%)	1 (20%)	6 (75%)	7 (54%)
	Mixture	0 (0%)	0 (0%)	0 (0%)	3 (60%)	0 (0%)	3 (23%)
**Preferred non-English language**							
	None	1 (17%)	1 (25%)	2 (20%)	1 (20%)	1 (13%)	2 (15%)
	Spanish	5 (83%)	3 (75%)	8 (80%)	4 (80%)	5 (63%)	10 (77%)
	Layman	0 (0%)	0 (0%)	0 (0%)	0 (0%)	1 (13%)	1 (8%)

#### Dental Patients

Evaluating potential HPV educational topics individually (Table 2), function of the vaccine in the body and vaccine schedule were also two of the most frequently patient identified important topics (92% each). Also aligning with provider preferences, most patients agreed and more frequently chose dentist (85%) and medical doctor (85%) as preferred HPV educators. The poster was the most frequently (92%) selected patient educational material of choice. Most adults (75%) selected real pictures as the preferred visual educational content; parents chose animated and real pictures (Table 2).

### Focus Groups to Review Existing HPV Educational Materials (*n* = 9; Data Not Shown)

#### Dental Providers

Two providers participated in the focus groups. Among the seven videos shown to providers, Team Maureen’s “Are you HUMAN?,” Louisiana State University’s “Shot by Shot,” and American Cancer Society (ACS)’s “HPV Campaign” videos were the most well-received with no negative comments provided. The Centers for Disease Control and Prevention (CDC)’s “Dr. Wolynn” video was the least liked with providers responding with phrases of “not enjoyable,” “not informative,” and “not relevant.”

Among reviewed print materials, providers reviewed the Team Maureen toolkit, consisting of a pamphlet, poster, talking tips, vaccine reminder card, and HPV vaccine policy statement, most favorably, and the American Academy of Pediatrics poster the least favorable. Providers asked for more sociodemographically diverse images and HPV vaccine details for both Team Maureen and I Vaccinate toolkits. With the I Vaccinate toolkit, including posters, pamphlet, fact sheets, vaccine safety sheet, and reframe the conversation guide, clinicians also requested additional citations/trusted sources of pro HPV vaccine professional groups, more details on HPV, and dental specific content.

#### Dental Patients

Seven patients participated in the focus groups. Among the four dental patient videos, the ACS’s “HPV Campaign” and Minnesota Department of Health’s “Steve’s Story” were the most liked. For these videos, patients agreed with more statements about overall, content, audio, and visual satisfaction than they did with the other two videos. Team Maureen’s “Are you HUMAN?” was the least liked with comments that provider-patient talking tips, trusted source(s), and common HPV vaccine concerns were missing topics. Among paper materials reviewed by patients, the Team Maureen toolkit, including the pamphlet, poster, and vaccine reminder card, was also preferred to I Vaccinate’s posters, pamphlet, fact sheets, and vaccine safety sheet. Patients wanted more image diversity with varying sociodemographic characteristics, HPV and HPV vaccine details, and citations/trusted sources in both toolkits. Patients also asked for a dental focus with I Vaccinate.

### Independent Reviews of Existing HPV Educational Materials (*n* = 6; Data Not Shown)

#### Dental Providers

Four dental providers completed independent reviews of educational materials. The ACS patient campaign was the highest rated video by this group. All hygienists rated eight of the overall, content, audio, and visual satisfaction review statements as agreeable; no dentist found the video interesting overall. Steve’s Survivor Story video was the second highest rated video by providers; no provider agreed that the video addressed “common concerns about oral HPV cancers.” The provider-focused ACS and CDC provider campaign videos were not rated as high (i.e., fewer “agreed” statements) as the patient-focused videos. No providers responded in the affirmative that the ACS-provider campaign video addressed “common concerns about the HPV vaccine” or that it reiterated the importance for 9–45-year-old Ohioans to get HPV vaccinated. The Dr. Wolynn video was rated the lowest by all, receiving the fewest positive statements.

For print materials, the Team Maureen toolkit was more positively reviewed for overall, content, visual, and/or audio satisfaction than I Vaccinate with all dentists agreeing on nine (of ten) review statements and hygienists on eight. Neutral feelings were more prevalent among providers regarding the I Vaccinate materials. The AAP poster was not as highly rated by providers as no providers agreed that the poster changed their mind about the HPV vaccine.

#### Dental Patients

Two patients completed the independent review survey. Among videos reviewed, Steve’s Survivor Story video was the highest rated. All patients rated all ten statements on overall, content, visual, and audio satisfaction as agreeable. The ACS patient campaign was the second highest rated video. All dental patients rated nine review statements as agreeable. For print materials, the Team Maureen toolkit was rated higher than I Vaccinate with all patients agreeing on all statements. Patients, like providers, expressed more neutral feelings towards the I Vaccinate materials.

## Discussion

The increase of oropharyngeal HPV cancer [2, 3] necessitates HPV and HPV vaccine educational efforts that could be implemented in dental offices [9, 10]. In the current study, dental providers and patients were more comfortable with HPV/oral cancer education and screening and less comfortable with HPV vaccine education and recommendation. Dental providers from the Appalachian region were not sharing any HPV educational materials and rarely engaging in educational conversations with dental patients. Among all groups, the ACS videos and Team Maureen toolkit received the most positive feedback, offering a basis for future dental clinic educational materials.

Our assessment of current efforts and future preferences for dental provider-patient HPV education was supported by previous research. As in the current study, prior studies have shown this lack of conceptual understanding of HPV, oropharyngeal cancer (OPC), and the HPV vaccine by dental providers [11–13, 18–22, 29, 30]. Other dental providers have also similarly concentrated more on OPC screenings and less on patient HPV educational conversations and HPV vaccine recommendations [11, 13, 18–24, 29, 31]. Previously, providers indicated toolkits increased their knowledge and confidence for discussing HPV with patients [14, 15].

Patients, namely parents, continue to be more receptive to HPV-related educational engagements than providers have initiated [21, 23, 24, 31]. Moreover, dental patient appropriate and community-targeted OPC and HPV vaccine educational materials are needed for Appalachian Ohio as suggested by this study and elsewhere in the USA as noted in previous studies [11–13, 23, 24]. Patients have also demonstrated increased HPV knowledge and vaccine intent when they were given materials (ex, comic books) to read [32].

A variety of dental-related HPV educational materials exist in different formats publicly. Until now, few resources have been formally investigated within end-users (e.g., Team Maureen in New England area, USA, dental professionals only) [14, 15] while most have not [16, 17]. Dental providers have had to locate and assess any materials independently as national dental professional organizations do not endorse any [9, 10, 12]. Therefore, the potential implications of the current study could be greater than previous investigations due to our awareness of some existing materials in one centralized location and the process for community involvement in material evaluation and modification. Such participation ensures greater material relevance and community buy-in. These educational materials can position providers with the knowledge to make strong HPV vaccine recommendations [6, 7, 33, 34] and patients with the information to make informed HPV vaccine decisions. Moreover, Appalachian Ohio (or region of study interest) HPV vaccine rates and, ultimately, their oral health could improve thereafter.

A major strength of the study was the in-depth critical review of existing HPV educational materials for dental visit use. Focus groups allowed for comprehensive feedback; the alternative approach of an independent review survey maximized participation. Reliance on multiple perspectives with both dental providers and patients, especially the uninvestigated HPV vaccine eligible 18–45 year old adult dental patients, meant increased material audience. Focus on a high-risk HPV and medically underserved population will allow for materials to be specifically targeted for these communities.

The study also had its limitations. First, the small sample size means results may not be fully representative of the communities investigated. Recruitment efforts utilized various channels of study promotion, but interest remained low. HPV social stigmas and the politicization of vaccines during the COVID-19 pandemic may have hindered study participation. Additionally, the time commitment of approximately 2 hours within the two-part (survey (15–30 min), focus group/independent review (90 min)) study may have also limited participation rates despite the advertised reasonable incentives ($10–$25 Amazon gift cards/study part). Independent review of materials through survey format meant some feedback was limited to Likert scale responses and the rarely used additional comments. The cross-sectional study design meant there was no opportunity for additional input post-editing. The specific study population, predominantly Appalachian Ohio women, limits generalizability, especially for the higher risk group of males, for currently reviewed HPV educational materials. There was also a disconnect in targeted areas—most providers were in urban areas while patients lived in rural residences. Future studies are needed to create other community-targeted HPV educational materials.

Our results need to be confirmed with a larger sample of similar Appalachian Ohio dental providers and patients. Existing HPV educational materials must be modified according to the recommendations, reviewed for additional feedback, and eventually evaluated for effectiveness in an intervention study. To date, only the Team Maureen toolkit has been investigated for dental provider effectiveness; the materials were demonstrated to be effective, but the study was restricted to a select northeastern population [14]. To reduce OPC rates nationally, additional interventions are needed to promote region-specific provider discussions and sharing of educational materials with patients to increase education and promotion of the HPV vaccine.

## Supplementary Information

Below is the link to the electronic supplementary material.

Supplementary Material 1

Supplementary Material 2

Supplementary Material 3

Supplementary Material 4

Supplementary Material 5
