# Supplementary material for: STEPS (Study To Examine Parent, Patient/Dental Provider Systems) to Prevent Human Papillomavirus (HPV)-Related Cancers: A Piloted Dental Patient and Provider Evaluation of Current and Future HPV Education
Source: J Cancer Educ. 2024 Jul 4;40(1):44–53. doi: 10.1007/s13187-024-02465-2 (PMC11846729; doi:10.1007/s13187-024-02465-2)
Supplement: Supplementary file 1 — Supplementary Material 1 [file 13187_2024_2465_MOESM1_ESM.pdf]

Article Title: STEPS To Prevent Human Papillomavirus (HPV)-related Cancers: A Piloted Dental Patient and Provider Evaluation of Current and Future HPV Education

Journal Name: Journal of Cancer Education

Author Names: Kelsey H. Jordan; Julie A. Stephens; Kaleigh Niles; Nina Hoffmeyer; Michael L. Pennell; Jill M. Oliveri; Electra D. Paskett

Corresponding Author: Kelsey H. Jordan

Affiliation: Division of Population Sciences, Comprehensive Cancer Center, The Ohio State University, Columbus, Ohio, USA

Email Address: kelsey.jordan@osumc.edu

Parent survey

Human Papillomavirus (HPV) & HPV Vaccine Knowledge Questions

Instructions: *Please select one option unless otherwise noted.*

1. **True or false: There are many types of HPV.**
   - ○ True
   - ○ False
   - ○ Unsure
   - ○ Prefer not to answer
2. **True or false: HPV is a common infection in the United States.**
   - ○ True
   - ○ False
   - ○ Unsure
   - ○ Prefer not to answer
3. **True or false: People may be infected with HPV and not know it.**
   - ○ True
   - ○ False
   - ○ Unsure
   - ○ Prefer not to answer
4. **What can the HPV vaccine offer protection against?**
   - ○ Only genital warts
   - ○ Only some cancers
   - ○ Genital warts and some cancers
   - ○ Neither genital warts nor any cancers
   - ○ AIDS
   - ○ Prefer not to answer

HPV, HPV Vaccine & Dental Visit Attitude Questions

HPV causes six cancers, most notably cervical cancer in women and mouth and throat cancers in men. The HPV vaccine has been approved since 2006 for children starting at age 9 and up to age 26.

Instructions: *Please respond with your level of agreement or disagreement with each of the following statements. Please mark only one response per statement.*

Question	Strongly Disagree	Disagree	Neither Agree nor Disagree	Agree	Strongly Agree	Prefer not to answer
**1. I would be comfortable with dental staff teaching <u>me</u> about HPV.**	○	○	○	○	○	○
**2. I would be comfortable with dental staff teaching <u>me</u> about the HPV vaccine.**	○	○	○	○	○	○
**3. I would like it if dental staff told <u>me</u> if my child(ren) needed the HPV vaccine.**	○	○	○	○	○	○
**4. I would be comfortable with dental staff teaching <u>my child(ren)</u> about the HPV vaccine.**	○	○	○	○	○	○
**5. I would be comfortable with dental staff telling <u>my children</u> they need the HPV vaccine.**	○	○	○	○	○	○
**6. I would be comfortable with dental staff checking <u>my child</u> for oral cancer.**	○	○	○	○	○	○

Current and Future HPV-based Concerns, Dental Visit Experiences

Instructions: *Items in this section are multiple choice. Please select one option unless otherwise noted.*

1. What concerns about <u>HPV</u> do you have for your child(ren)? (Mark all that apply.)

- ○ Catching HPV by having sex
- ○ Spreading HPV by having sex
- ○ Risk of getting genital warts
- ○ Risk of cancer
- ○ Everything (I don't know anything about HPV.)
- ○ Other (please specify): ____
- ○ I have no concerns
- ○ Prefer not to answer

2. Are your concerns about <u>HPV</u> different if your child(ren) are/were age 11-12 years old versus 13-17 years old?

- ○ Yes
  Please explain: 11-12 year old HPV concerns: ______________________________
  13-17 year old HPV concerns: ______________________________
- ○ No
- ○ Prefer not to answer

3. What concerns do you have about getting the HPV vaccine for your child(ren)? (Mark all that apply.)

- ○ Not having the HPV vaccine information I need to make a decision
- ○ Child(ren) becoming sexually active earlier in life
- ○ Pain that the HPV vaccine may cause my child(ren)
- ○ Vaccine cost
- ○ Vaccine safety (potential side effects)
- ○ Length of vaccine's protection for child(ren)
- ○ Not having a HPV vaccine recommendation from my child(ren)'s healthcare provider
- ○ Family and/or friends not liking the HPV vaccine
- ○ My child(ren) not wanting the HPV vaccine
- ○ Cultural concerns
- ○ Religious objections
- ○ Other (please specify): ____
- ○ I have no concerns
- ○ Prefer not to answer

4. Would your concerns about the HPV vaccine be different if your child(ren) are/were 11-12 years old versus 13-17 years old?

- ○ Yes
  Please explain: 11-12 year old HPV vaccine concerns: ________________________
  13-17 year old HPV vaccine concerns: ________________________
- ○ No
- ○ Prefer not to answer

5. Has/have your child(ren) ever been checked for oral cancer by a dental staff member?

- ○ Yes (proceed to question #6)
- ○ No (proceed to question #8)
- ○ Unsure (proceed to question #8)
- ○ Prefer not to answer (proceed to question #8)

6. How often is/are your child(ren) being checked for oral cancer by a dental staff member?

- ○ Only at one visit
- ○ Some visits
- ○ Every visit
- ○ Unsure
- ○ Prefer not to answer

7. Does/did the dental staff explain the oral cancer exam to you and/or your child(ren)?

- ○ Yes

- ○ No
- ○ Unsure
- ○ Prefer not to answer

8. Does anyone talk to you and/or your child(ren) about HPV topics at their dental visit(s)?

- ○ Yes
  - **If yes, please specify who talks about HPV with you and/or your child(ren). (Mark all that apply.)**
    - Dentist(s)
    - Hygienists
    - Dental assistants
    - Other dental office staff (please specify): ________________________
- ○ No
- ○ Unsure
- ○ Prefer not to answer

9. Is information about the HPV vaccine <u>currently</u> being shared with you and/or your child(ren) at their dental visits?

- ○ Yes
  - If yes, please specific <u>all</u> formats that apply. **(Mark all that apply.)**
    - Conversations
    - Posters in lobby/patient rooms
    - Brochures
    - Fact sheets
    - Newsletters
    - Videos
    - Other (please specify): ______________________________________
- ○ No
- ○ Unsure
- ○ Prefer not to answer

10. Please tell us how important each of the following topics are to parents <u>needing</u> to know about HPV. (Mark one answer per statement.)

HPV/oral cancer disease descriptions

Very important somewhat important neutral somewhat unimportant not important at all

Who can get HPV

Very important somewhat important neutral somewhat unimportant not important at all

Oral cancer self-exam steps

Very important somewhat important neutral somewhat unimportant not important at all

HPV cancer statistics

Very important somewhat important neutral somewhat unimportant not important at all

How the HPV vaccine works
Very important somewhat important neutral somewhat unimportant not important at all

HPV vaccine costs
Very important somewhat important neutral somewhat unimportant not important at all

At what ages the HPV vaccine is given
Very important somewhat important neutral somewhat unimportant not important at all

The number of HPV vaccine doses needed
Very important somewhat important neutral somewhat unimportant not important at all

Child-friendly HPV and HPV vaccine talking points
Very important somewhat important neutral somewhat unimportant not important at all

- ○ Prefer not to answer

11. Are there any other HPV-related topics that parents need to know about?

- ○ Yes
  - ○ If yes, please specify: ____
- ○ No
- ○ Prefer not to answer

12. In education materials coming from the dentist's office, who should teach parents about HPV? (Mark all that apply.)

- ○ Public health professional
- ○ Medical doctor
- ○ Nurse
- ○ Dentist
- ○ Hygienist
- ○ Dental assistant
- ○ Oral cancer patient
- ○ Other (please specify): ________________________________
- ○ Prefer not to answer

13. In what format(s) should HPV information be given to parents? (Mark all that apply.)

- ○ Posters in lobby or exam rooms
- ○ Brochures
- ○ Fact sheets
- ○ Talking tips
- ○ Newsletters
- ○ Additional resources lists

- ○ Videos
- ○ Other (please specify): ____
- ○ Prefer not to answer

14. How should statistics about HPV be given to parents? (Mark all that apply.)

- ○ Stories
- ○ Bar charts
- ○ Numbers/percentages
- ○ Other (please specify): ___________________________________
- ○ Prefer not to answer

Now, we would like to know your opinions on how HPV education materials should look to parents of young dental patients.

15. Do you have an opinion on the colors used in HPV education materials for parents of 9-17 year old dental patients?

- ○ Yes
    - o If yes, please specify.
        - Colors to include: ____
        - Colors to exclude: ____
- ○ No

16. Do you have an opinion on how text will appear (e.g., bullet points, sentences/paragraphs, etc.) in the HPV education materials for parents of 9-17 year old dental patients?

- ○ Yes
    - o If yes, please choose one of the following:
        - Bullet points
        - Sentences/paragraphs
        - Other (please specify): _____________________________________
- ○ No
- ○ Prefer not to answer

17. Do you have an opinion on the types of pictures (e.g., real vs. animated) that will be used in the HPV education materials for parents of 9-17 year old dental patients?

- ○ Yes
    - o If yes, please choose one of the following:
        - Animated/cartoon
        - Real/life-like images
        - Mixture of animated & real images

- Other (please specify): ____

- No
- Prefer not to answer

18. In your opinion, aside from English, do the HPV education materials for parents of 9-17 year old dental patients need to be translated to another language or other languages?

- Yes
  - If yes, please choose one of the following options:
    - Spanish
    - Other (please specify): ____
- No
- Prefer not to answer

Adult survey

Human Papillomavirus (HPV) & HPV Vaccine Knowledge Questions

Instructions: *Please select one option per question.*

1. **True or false: There are many types of HPV.**
   - True
   - False
   - Unsure
   - Prefer not to answer
2. **True or false: HPV is a common infection in the United States.**
   - True
   - False
   - Unsure
   - Prefer not to answer
3. **True or false: People may be infected with HPV and not know it.**
   - True
   - False
   - Unsure
   - Prefer not to answer
4. **What can the HPV vaccine offer protection against?**
   - Only genital warts
   - Only some cancers
   - Genital warts and some cancers
   - Neither genital warts nor any cancers
   - AIDS
   - Prefer not to answer

HPV, HPV Vaccine & Dental Visit Attitude Questions

HPV causes six cancers, most notably cervical cancer in women and mouth and throat cancers in men. The HPV vaccine has been approved since 2006 for 9 to 21 year old males and 9 to 26 year old females.

The ACIP (Advisory Committee on Immunization Practice) has made some changes to who can receive the HPV vaccine. There were two changes:

First, men can now obtain catch-up vaccinations through age 26 years old. Previously, for average risk men, it was only recommended that they be vaccinated through age 21 years old.

The second change was that adult men and women ages 27 – 45 years old can now be vaccinated if informed of the risks and benefits in a conversation with their health care provider that uses shared decision making.

Instructions: *Please respond with your level of agreement or disagreement with each of the following statements. Please mark only one response per statement.*

**Question**	Strongly Disagree	Disagree	Neither Agree nor Disagree	Agree	Strongly Agree	Prefer not to answer
**1. I would be comfortable with dental staff teaching me about HPV.**	○	○	○	○	○	○
**2. I would be comfortable with dental staff teaching me about oral cancer.**	○	○	○	○	○	○
**3. I would be comfortable with dental staff checking me for oral cancer.**	○	○	○	○	○	○
**4. I would be comfortable with dental staff teaching me about the HPV vaccine.**	○	○	○	○	○	○
**5. I would like it if dental staff told me that I needed to get the HPV vaccine.**	○	○	○	○	○	○

Current and Future HPV-based Concerns, Dental Visit Experiences

Instructions: *Items in this section are multiple choice. Please select one option unless otherwise noted.*

1. **What concerns you about getting HPV? (Mark all that apply.)**
   - ○ Catching HPV by having sex
   - ○ Spreading HPV by having sex
   - ○ Risk of genital warts
   - ○ Risk of cancer
   - ○ Everything (I don't know anything about HPV.)
   - ○ Other (please specify): ____
   - ○ I have no concerns
   - ○ Prefer not to answer
2. **What concerns do you have about getting the HPV vaccine? (Mark all that apply.)**
   - ○ Not having the HPV vaccine information I need to make a decision
   - ○ Pain that the HPV vaccine may cause me
   - ○ Vaccine cost

- Vaccine safety (potential side effects)
- Length of vaccine's protection
- Not having a HPV vaccine recommendation from my healthcare provider(s)
- Family and/or friends not liking the HPV vaccine
- Cultural concerns
- Religious objections
- Other (please specify): ______________________________
- I have no concerns
- Prefer not to answer

3. Have you ever been checked for oral cancer by a dental staff member?

- Yes (proceed to question #4)
- No (proceed to question #6)
- Unsure (proceed to question #6)
- Prefer not to answer (proceed to question #6)

4. How often have you been checked for oral cancer by your dental office?

- Only at one visit
- Some visits
- Every visit
- Unsure
- Prefer not to answer

5. Does/did the dental staff explain the oral cancer exam to you?

- Yes
- No
- Unsure
- Prefer not to answer

6. Does anyone talk to you about HPV topics at your dental visits?

- Yes
  - **If yes, please specify who talks about HPV with you. (Mark all that apply.)**
    - Dentist(s)
    - Hygienists
    - Dental assistants
    - Other dental office staff (please specify): ____________________
- No
- Unsure
- Prefer not to answer

7. Is information about the HPV vaccine currently being shared with you at your dental visits?

- ○ Yes
  - If yes, please specific all formats that apply. **(Mark all that apply.)**
    - Conversations
    - Posters in lobby/patient rooms
    - Brochures
    - Fact sheets
    - Newsletters
    - Videos
    - Other (please specify): ______________________________________
- ○ No
- ○ Unsure
- ○ Prefer not to answer

8. Please tell us how important each of the following topics are for adults needing to know about HPV. (Mark one answer per statement.)

HPV/oral cancer disease descriptions
Very important somewhat important neutral somewhat unimportant not important at all

Who can get HPV
Very important somewhat important neutral somewhat unimportant not important at all

Oral cancer self-exam steps
Very important somewhat important neutral somewhat unimportant not important at all

HPV cancer statistics
Very important somewhat important neutral somewhat unimportant not important at all

How the HPV vaccine works
Very important somewhat important neutral somewhat unimportant not important at all

HPV vaccine costs
Very important somewhat important neutral somewhat unimportant not important at all

At what ages the HPV vaccine is given
Very important somewhat important neutral somewhat unimportant not important at all

The number of doses of the HPV vaccine people need
Very important somewhat important neutral somewhat unimportant not important at all

- ○ Prefer not to answer

9. Are there any other HPV-related topics that adults need to know about?

- ○ Yes
  - If yes, please specify: ____

- ○ No
- ○ Prefer not to answer

10. In education materials coming from the dentist's office, who should teach adults about HPV? (Mark all that apply.)

- ○ Public health professional
- ○ Medical doctor
- ○ Nurse
- ○ Dentist
- ○ Hygienist
- ○ Dental assistant
- ○ Oral cancer patient
- ○ Other (please specify): ________________________________
- ○ Prefer not to answer

11. In what format(s) should HPV information be given to adults? (Mark all that apply.)

- ○ Posters in lobby or exam rooms
- ○ Brochures
- ○ Fact sheets
- ○ Talking tips
- ○ Newsletters
- ○ Additional resources lists
- ○ Videos
- ○ Other (please specify): ____
- ○ Prefer not to answer

12. How should statistics about HPV be given to adults? (Mark all that apply.)

- ○ Stories
- ○ Bar charts
- ○ Numbers/percentages
- ○ Other (please specify): _________________________________
- ○ Prefer not to answer

Now, we would like to know your opinions on how HPV education materials should look to adult dental patients.

13. Do you have an opinion on the colors used in HPV education materials for adult dental patients?

- ○ Yes
  - o If yes, please specify.

- Colors to include: ____
- Colors to exclude: ____

○ No

○ Prefer not to answer

14. Do you have an opinion on how text will appear (e.g., bullet points, sentences/paragraphs, etc.) in the HPV education materials for adult dental patients?

○ Yes
- If yes, please choose one of the following:
  - Bullet points
  - Sentences/paragraphs
  - Other (please specify): ___________________________________

○ No

○ Prefer not to answer

15. Do you have an opinion on the types of pictures (e.g., real vs. animated) that will be used in the HPV education materials for adult dental patients?

○ Yes
- If yes, please choose one of the following:
  - Animated/cartoon
  - Real/life-like images
  - Mixture of animated & real images
  - Other (please specify): _____________________________________

○ No

○ Prefer not to answer

16. In your opinion, aside from English, do the HPV education materials for adult dental patients need to be translated to another language or other languages?

○ Yes
- If yes, please choose one of the following:
  - Spanish
  - Other (please specify): _____________________________________

○ No

○ Prefer not to answer

Dental Provider survey

Current and Future HPV-based Concerns, Dental Visit Experiences

Instructions: *Items in this section are multiple choice. Please select one option unless otherwise noted.*

1. **Do you ever perform oral cancer screenings on your patients?**
   - ○ Yes (proceed to question #2)
   - ○ No (proceed to question #3)
   - ○ Prefer not to answer (proceed to question #3)
2. **What age groups of your patients receive oral cancer screenings? (Mark all that apply.)**
   - ○ Young children (i.e., ≤ 8 years old)

     How often do you perform oral screenings with young children (i.e., ≤ 8 years old)? (Mark one answer.)

     Always, most of the time, sometimes, rarely

     I am comfortable doing oral cancer screenings on young children (i.e., ≤ 8 years old). (Mark only one.)

     Strongly disagree, disagree, neither agree/disagree, agree, strongly agree

   - ○ Adolescents (i.e., 9-17 years old)

     Which ages?

     9-10; 11-12; 13-17 (Mark all that apply.)

     How often do you perform oral screenings with adolescents (i.e., 9-17 years old)? (Mark one answer.)

     Always, most of the time, sometimes, rarely

     I am comfortable doing oral cancer screenings on adolescents (i.e., 9-17 years old). (Mark only one.)

     Strongly disagree, disagree, neither agree/disagree, agree, strongly agree

   - ○ Young adults (i.e., 18-45 years old)

     Which ages?

     18-26; 27-45 (Mark all that apply.)

     How often do you perform oral screenings with young adults (i.e., 18-45 years old)? (Mark one answer.)

     Always, most of the time, sometimes, rarely

     I am comfortable doing oral cancer screenings on young adults (i.e., 18-45 years old). (Mark only one.)

     Strongly disagree, disagree, neither agree/disagree, agree, strongly agree

- Older adults (i.e., ≥ 46 years old)

  How often do you perform oral screenings with older adults (i.e., ≥ 46 years old)? (Mark one answer.)

  Always, most of the time, sometimes, rarely

  I am comfortable doing oral cancer screenings on older adults (i.e., ≥ 46 years old). (Mark only one.)

  Strongly disagree, disagree, neither agree/disagree, agree, strongly agree

- Prefer not to answer

3. Do you provide any of your patients with HPV vaccine information?

- Yes (proceed to question #4)
- No (proceed to question #7)

  Why not? (Mark all that apply.)

  Lack of knowledge, lack of materials to share, sensitive topic, other (please specify): ___________

- Prefer not to answer (proceed to question #7)

4. Which age groups receive HPV vaccine information in your office? (Mark all that apply.)

- Young children (i.e., ≤ 8 years old)
- Adolescents (i.e., 9-17 years old)
- Parents of child dental patients (i.e., ≤ 17 years old)
- Young adults (i.e., 18-45 years old)
- Prefer not to answer

5. In what format(s) is HPV vaccine information being shared with your patients? (Mark all that apply.)

- Conversations
- Posters
- Brochures
- Fact sheets
- Newsletters
- Videos
- Other (please specify): ____
- Prefer not to answer

6. Who provides HPV vaccine information to your patients? (Mark all that apply.)

- Dentist(s)
- Hygienists
- Dental assistants
- Other dental office staff: ________________________________

○ Prefer not to answer

7. Please tell us how important each of the following HPV-related topic(s) is for dental providers to know when talking with their patients about HPV. (Mark one answer per statement.)

HPV/oropharyngeal cancer disease descriptions
Very important somewhat important neutral somewhat unimportant not important at all

At-risk populations
Very important somewhat important neutral somewhat unimportant not important at all

Oropharyngeal cancer screening steps
Very important somewhat important neutral somewhat unimportant not important at all

HPV cancer statistics
Very important somewhat important neutral somewhat unimportant not important at all

HPV vaccine function
Very important somewhat important neutral somewhat unimportant not important at all

HPV vaccine costs
Very important somewhat important neutral somewhat unimportant not important at all

HPV vaccine schedule
Very important somewhat important neutral somewhat unimportant not important at all

HPV vaccine ages
Very important somewhat important neutral somewhat unimportant not important at all

Child-friendly HPV and HPV vaccine talking points
Very important somewhat important neutral somewhat unimportant not important at all

○ Prefer not to answer

8. Are there any other HPV-related topics that dental providers need to know to better assist them in talking with their patients about HPV?

○ Yes
  - If yes, please specify: ____

○ No

○ Prefer not to answer

9. What health professional(s) should be depicted in dental providers' HPV educational materials? (Mark all that apply.)

○ Public health professional

○ Medical doctor

○ Nurse

- ○ Dentist
- ○ Hygienist
- ○ Dental assistant
- ○ Oropharyngeal cancer patient
- ○ Other (please specify): ________________________________
- ○ Prefer not to answer

10. In what format(s) should HPV educational materials be presented to dental providers? (Mark all that apply.)

- ○ Presentations/group meetings
- ○ Posters
- ○ Brochures
- ○ Fact sheets
- ○ Talking points
- ○ Newsletters
- ○ Additional resources lists
- ○ Videos
- ○ Other: ____
- ○ Prefer not to answer

11. How should HPV statistics be presented to dental providers? (Mark all that apply.)

- ○ Stories
- ○ Bar charts
- ○ Numerical values (e.g., percentages, rates)
- ○ Other: ________________________________
- ○ Prefer not to answer

Now, we would like to know your visual preferences for dental provider HPV education materials.

12. Do you have a preference on the colors used in dental provider HPV education materials?

- ○ Yes
  - o If yes, please specify.
    - Colors to include: ____
    - Colors to exclude: ____
- ○ No
- ○ Prefer not to answer

13. Do you have a preference on how text will appear (e.g., bulleted lists, sentences/paragraphs, etc.) in the dental provider HPV education materials?

- ○ Yes
  - o If yes, please choose one of the following:
    - Bullet points
    - Sentences/paragraphs
    - Other (please specify): ___________________________________
- ○ No
- ○ Prefer not to answer

14. Do you have a preference on the types of pictures (e.g., real vs. animated) that will be used in dental provider HPV education materials?

- ○ Yes
  - o If yes, please choose one of the following:
    - Animated/cartoon
    - Real/life-like images
    - Mixture of animated & real images
    - Other (please specify): _____________________________________
- ○ No
- ○ Prefer not to answer

15. In your opinion, aside from English, do the dental provider HPV education materials need to be translated to another language or other languages?

- ○ Yes
  - o If yes, please choose one of the following options:
    - Spanish
    - Other (please specify): _____________________________________
- ○ No
- ○ Prefer not to answer

HPV & HPV Vaccine Knowledge Questions

Instructions: *Please respond with your level of agreement or disagreement to each of the following statements. Please mark only one response per statement.*

**Question**	Strongly Disagree	Disagree	Neither Agree nor Disagree	Agree	Strongly Agree	Prefer not to answer
**1. The human papillomavirus (HPV) always has visible signs or symptoms.**	○	○	○	○	○	○

**2. HPV usually goes away without needing any treatment.**	○	○	○	○	○	○
**3. Most sexually active people will get HPV at some point in their lives.**	○	○	○	○	○	○
**4. The tongue is the primary head and neck cancer site associated with HPV.**	○	○	○	○	○	○

Instructions: *Please select one option per statement.*

5. True or false: The HPV vaccine is only recommended for females.

- ○ True
- ○ False
- ○ Unsure
- ○ Prefer not to answer

6. True or false: The HPV vaccine prevents all oral cancers.

- ○ True
- ○ False
- ○ Unsure
- ○ Prefer not to answer

HPV, HPV Vaccine & Dental Visit Attitude Questions

HPV causes six cancers, most notably cervical cancer in women and mouth and throat cancers in men. The HPV vaccine has been approved since 2006 for 9 to 21 year old males and 9 to 26 year old females.

The ACIP (Advisory Committee on Immunization Practice) has made some changes to who can receive the HPV vaccine. There were two changes:

First, men can now obtain catch-up vaccinations through age 26 years old. Previously, for average risk men, it was only recommended that they be vaccinated through age 21 years old.

The second change was that adult men and women ages 27 – 45 years old can now be vaccinated if informed of the risks and benefits in a conversation with their health care provider that uses shared decision making.

Instructions: *Please respond with your level of agreement or disagreement with each of the following statements. Please mark only one response per statement.*

**Question**	Strongly Disagree	Disagree	Neither Agree nor Disagree	Agree	Strongly Agree	Prefer not to answer
**1. My community is concerned about contracting HPV.**	○	○	○	○	○	○
**2. My community is concerned about getting oropharyngeal cancer.**	○	○	○	○	○	○
**3. I think it is appropriate for dental providers to screen children 9-17 years old for oropharyngeal cancer.**	○	○	○	○	○	○
**4. I think it is appropriate for dental providers to teach children 9-17 years old about the HPV vaccine.**	○	○	○	○	○	○
**5. I think it is appropriate for dental providers to recommend the HPV vaccine to children 9-17 years old.**	○	○	○	○	○	○
**6. I think it is appropriate for dental providers to teach parents of children 9-17 years old about the HPV vaccine.**	○	○	○	○	○	○
**7. I think it is appropriate for dental providers to recommend to parents of children 9-17 years old that they get their children vaccinated against HPV.**	○	○	○	○	○	○
**8. In consultation with their doctor, I think men and women up to age 45 years should be eligible for the HPV vaccine.**	○	○	○	○	○	○
**9. I think it is appropriate for dental providers to teach 18-45 year old adults about HPV.**	○	○	○	○	○	○
**10. I think it is appropriate for dental providers to teach 18-45 year old adults about oropharyngeal cancer.**	○	○	○	○	○	○
**11. I think it is appropriate for dental providers to screen 18-45 year olds adults for oropharyngeal cancer.**	○	○	○	○	○	○
**12. I think it is appropriate for dental providers to teach 18-45 year old adults about the HPV vaccine.**	○	○	○	○	○	○
**13. I think it is appropriate for dental providers to recommend the HPV vaccine to 18-45 year old adults.**	○	○	○	○	○	○
**14. I think my 18-45 year old patients are/would be receptive to HPV vaccine education.**	○	○	○	○	○	○
