# Supplementary material for: STEPS (Study To Examine Parent, Patient/Dental Provider Systems) to Prevent Human Papillomavirus (HPV)-Related Cancers: A Piloted Dental Patient and Provider Evaluation of Current and Future HPV Education
Source: J Cancer Educ. 2024 Jul 4;40(1):44–53. doi: 10.1007/s13187-024-02465-2 (PMC11846729; doi:10.1007/s13187-024-02465-2)
Supplement: Supplementary file 2 — Supplementary Material 2 [file 13187_2024_2465_MOESM2_ESM.pdf]

Article Title: STEPS To Prevent Human Papillomavirus (HPV)-related Cancers: A Piloted Dental Patient and Provider Evaluation of Current and Future HPV Education

Journal Name: Journal of Cancer Education

Author Names: Kelsey H. Jordan; Julie A. Stephens; Kaleigh Niles; Nina Hoffmeyer; Michael L. Pennell; Jill M. Oliveri; Electra D. Paskett

Corresponding Author: Kelsey H. Jordan

Affiliation: Division of Population Sciences, Comprehensive Cancer Center, The Ohio State University, Columbus, Ohio, USA

Email Address: kelsey.jordan@osumc.edu

Team Maureen Dental Toolkit

Pamphlet—pg 1

ORAL, HEAD & NECK SELF EXAM GUIDE

Check your mouth and neck each month. Talk to your dentist or doctor if you notice a lump or have any other changes that last over 2 weeks.

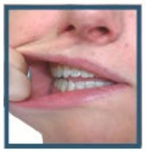

Use a mirror or flashlight to look at your cheeks, tongue, gums, back of the throat, and the roof of your mouth.

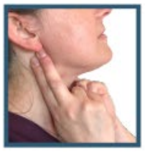

Feel for lumps behind your ears and down to your neck on both sides.

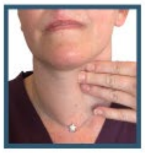

Feel along your neck all the way down to your collarbone.

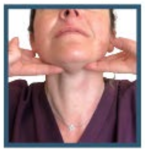

Lift your chin and feel upward to check underneath your jaw.

PREVENT CANCER CHECKLIST

- [x] Get Vaccinated!
- [ ] See your doctor and dentist regularly
- [ ] Check your mouth each month for any changes
- [ ] Stop smoking & limit drinking
- [ ] Talk to your doctor about cervical cancer screening

TEAM MAUREEN — JOINING TOGETHER TO END CERVICAL CANCER

For more information on HPV, visit:
cdc.gov/hpv
mahpvcoalition.org
teammaureen.org

PREVENTING CANCER at the Dentist!

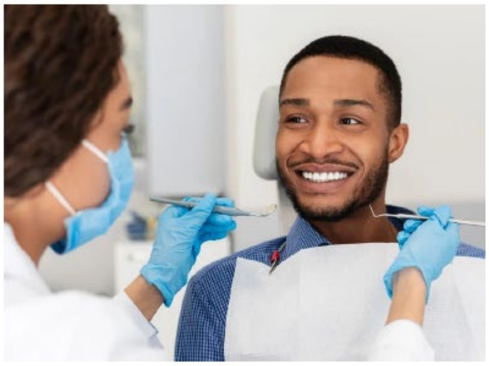

Stop Cancer Before it Starts

Talk to your dentist about the HPV vaccine today.

MASSACHUSETTS HPV COALITION

Pamphlet—pg 2

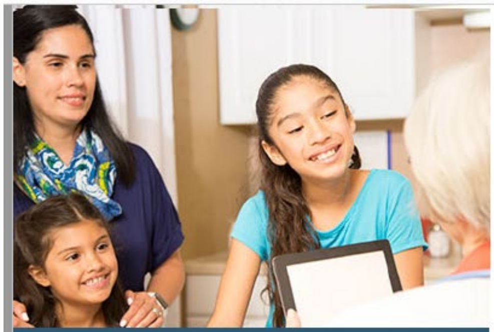

THE CANCER-PREVENTING VACCINE

The HPV vaccine provides protection from the most cancer-causing types of HPV. It is recommended for everyone between the ages of 9 to 26.

The best time to get the HPV vaccine is between ages 9 and 12. This is because children have a stronger immune response.

The vaccine is given in 2 or 3 doses over several months. Children age 14 or younger need 2 shots. After age 14, an extra catch-up shot is needed to be fully protected.

The HPV vaccine is very safe. Over 120 million doses of the HPV vaccine have been given since 2006. Like any other vaccine, the most common side effect is soreness at the injection site. Talk to your doctor if you have any severe allergies.

WHAT IS HUMAN PAPILLOMAVIRUS (HPV)?

Human Papilloma Virus (HPV) is a very common virus that spreads from skin-to-skin contact. Almost everyone will have HPV at some point in their life. HPV usually has no symptoms, so most people with HPV don't know they have it. Most cases of HPV go away on their own, but other cases can go on to cause cancer.

HPV can cause several types of cancer, including cervical and oropharyngeal cancer. While cervical cancer impacts women or people with a cervix, over 80% of oropharyngeal cancer cases are in men.

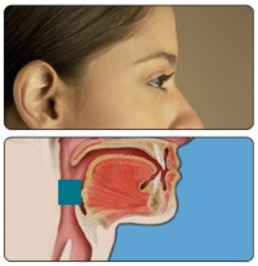

The oropharynx is the area in blue at the back of the tongue and throat.

The oropharynx is the back of the tongue and throat. Symptoms of oropharyngeal cancer include:

- Hoarseness or change of voice
- Sore throat that won't go away
- Earaches
- Swelling or lump in the neck
- Pain or difficulty swallowing
- Unexpected weight loss
- Sores or bumps in the mouth that last over 2 weeks

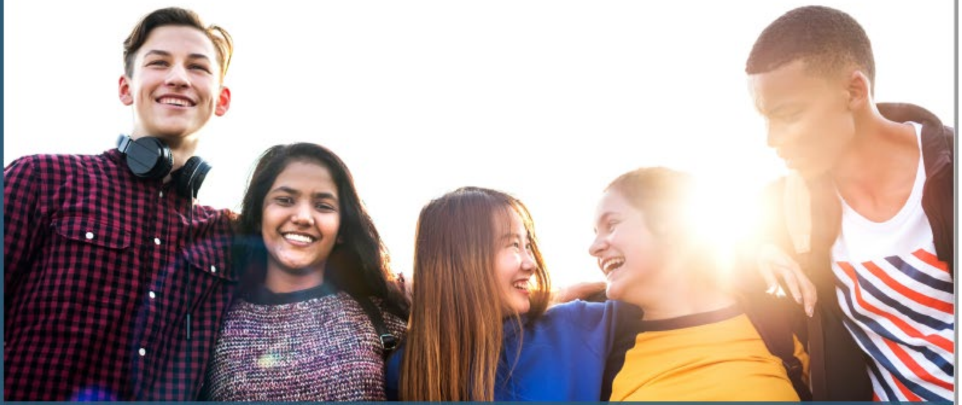

The HPV vaccine is for **everyone.**

Poster—11x17

PREVENTING CANCER
At the Dentist!

What is HPV?

Human Papilloma Virus (HPV) is a very common virus that spreads from skin-to-skin contact. **Almost everyone will have HPV at some point in their life.**

HPV usually has no symptoms, so most people with HPV don't know they have it. Most cases of HPV go away on their own, but other cases can go on to cause cancer.

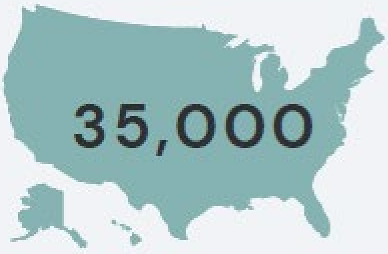

HPV causes over 35,000 cases of cancer in the US every year. About 21,000 cases are found in women and 14,000 are found in men.

HPV & Cancer

HPV causes 6 types of cancer. The most common are oropharyngeal and cervical cancer. The oropharynx is the area in the back of the tongue and throat.

80% of oropharyngeal cancer cases are found in men. Cervical cancer is found in women and people with a cervix.

The vaccine is given in **2-3 doses** over several months. If you are 14 or younger, you need 2 shots. After age 14, you will need an extra catch-up shot to be fully protected.

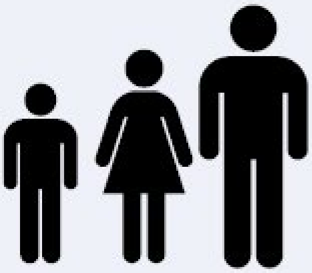

The vaccine is for everyone ages 9 to 26.

The HPV vaccine protects you or your child from the most cancer-causing strains of HPV and 90% of genital warts.

The best time to get the HPV vaccine is between ages 9 and 12. This is because children have a stronger immune response.

Prevent Cancer Checklist:

- Get Vaccinated!
- Check your mouth each month for any changes.
- See your doctor and dentist regularly.
- Talk to your doctor about cancer screening.

Talking Tips—Pg 1

HPV AT THE DENTIST

Parent Talking Tips

Start the Conversation: "Is your child up to date on the HPV vaccine? It prevents 6 types of cancer, including oropharyngeal cancer. Make sure to ask your child's doctor at your next visit."

Parents May Ask	Your Answer
Why does my child need the HPV vaccine?	HPV is extremely common – **most people will have HPV during their life.** By vaccinating your child, you can protect them from several types of cancer.
Can't we wait until they are older?	**The best time to get the HPV vaccine is between ages 9 and 12.** This is because children have a stronger immune response. After age 14, they will need an extra catch-up shot to be fully protected.
Does the vaccine really work?	**Yes,** studies continue to prove the HPV vaccine works extremely well. We have seen a significant decrease in the number of infections and cervical precancer in young people since the vaccine was introduced in 2006.
I thought the HPV vaccine was only for girls?	**The vaccine was first recommended for girls, but is now recommended for all children.** The vaccine prevents both cervical and oropharyngeal (throat/ back of tongue) cancer. In fact, oropharyngeal cancer is now more common than cervical cancer, and is more likely to impact men.
How much does the vaccine cost?	Like other childhood vaccines in Massachusetts, the HPV vaccine is free. Talk to your doctor or call your health insurance to find a provider.
I'm worried about the safety of the vaccine. Do you think it is safe?	Yes, the HPV vaccination is very safe. Like any other shot, the most common side effect is soreness at the injection site. Over 120 million doses of the HPV vaccine have been given and no serious safety concerns have been found. **Talk to your doctor if you still have questions.**

Talking Tips—Pg 2

HPV AT THE DENTIST

Age 18-26 Talking Tips

Start the Conversation: "Have you gotten the HPV vaccine? It prevents 6 types of cancer, including oropharyngeal cancer. Ask your doctor about the vaccine at your next visit."

Parents May Ask	Your Answer
What is Human Pappiloma Virus (HPV)?	HPV is an extremely common virus that spreads through sexual and intimate contact. **Most of us will have HPV at some point in our lives.** HPV can cause genital warts and 6 types of cancer, including oropharyngeal cancer.
Am I too old for the HPV vaccine?	It is most effective when given between the ages 9-12 but **the vaccine is recommended for everyone up to age 26.**
Can I still get the vaccine if I already have HPV?	**Yes.** The vaccine can protect you from other types in the future.
Can I still get HPV if I am vaccinated?	**Unfortunately, yes.** There are over 100 different strains, or types, of HPV. The vaccine protects you from 9 strains that are responsible for about 90% of genital warts and HPV-related cancers.
Is there a way to test for oral HPV?	**Not yet.** Researchers are working on developing a test, but so far they are unreliable.
I'm worried about the safety of the vaccine. Do you think it is safe?	**Yes, the HPV vaccination is very safe.** Soreness at the injection site is common, but over 120 million doses of the HPV vaccine have been given and no serious safety concerns have been found. **Talk to your doctor if you still have questions.**

Talking Tips—Pg 3

HPV AT THE DENTIST

Age 27+ Talking Tips

Start the Conversation: "Did you get the HPV vaccine as a child or young adult? It prevents 6 types of cancer including oropharyngeal cancer."

Parents May Ask	Your Answer
Am I too old for the HPV vaccine?	**Talk to your doctor to see if you would benefit from getting vaccinated.** The vaccine is approved up to age 45, but It is not usually recommended after age 26 because most adults have already been exposed to HPV by this time.
What is oropharyngeal cancer?	**The oropharynx is the area at the back of your throat.** It includes your tonsils, the base of your tongue and the back of the roof of your mouth. **Cancer in this area is usually caused by HPV.**
How common is HPV-related oropharyngeal cancer?	**HPV causes almost 20,000 cases of oropharyngeal cancer every year and the number of cases is increasing each year.** Oropharyngeal cancer is now the most common HPV-related cancer.
What are the symptoms of oropharyngeal cancer?	Symptoms include hoarseness, pain or difficulty swallowing, pain while chewing, a lump in the neck, a feeling of a persistent lump in the throat, change in voice, or non-healing sores on the neck.
Who is at risk of HPV-related oropharyngeal cancer?	**Anyone can get oropharyngeal cancer, but it is more common in men than women.** It is usually transmitted through oral sex. Having multiple sex partners can increases your risk. Smoking and drinking may also increase your risk of oropharyngeal cancer.
How serious is HPV-related oropharyngeal cancer?	Oropharyngeal cancer is usually treated with a combination of chemotherapy, radiation, and surgery. **The 5-year survival rate is 85-90%.** This is much higher than oropharyngeal cancers that aren't caused by HPV.

Vaccine Reminder Cards

Rx

Date ___________

HPV Vaccine Reminder

__________________ *is due for the cancer-preventing HPV Vaccine. Call their doctor to schedule an appointment!*

MASSACHUSETTS HPV COALITION

Rx

Date ___________

HPV Vaccine Reminder

__________________ *is due for the cancer-preventing HPV Vaccine. Call their doctor to schedule an appointment!*

MASSACHUSETTS HPV COALITION

Rx

Date ___________

HPV Vaccine Reminder

__________________ *is due for the cancer-preventing HPV Vaccine. Call their doctor to schedule an appointment!*

MASSACHUSETTS HPV COALITION

Rx

Date ___________

HPV Vaccine Reminder

__________________ *is due for the cancer-preventing HPV Vaccine. Call their doctor to schedule an appointment!*

MASSACHUSETTS HPV COALITION

HPV Vaccine Policy Statement—Pg 1

Policy on Human Papilloma Virus Vaccinations

Revised
2020

How to Cite: American Academy of Pediatric Dentistry. Policy on human papilloma virus vaccinations. The Reference Manual of Pediatric Dentistry. Chicago, Ill.: American Academy of Pediatric Dentistry; 2020:102-3.

Purpose

The American Academy of Pediatric Dentistry (**AAPD**) recognizes there is a link between human papilloma virus (**HPV**) and development of oral pharyngeal cancers. The purpose of this policy is to provide a perspective on dental provider's role in discussing oral cancers and their associations with HPV, and HPV vaccination for age-appropriate patients.

Methods

This policy was developed by the Council on Clinical Affairs and adopted in 2017.[1] This revision is based on a review of current dental and medical literature. An electronic search was conducted using the PubMed®/MEDLINE database using the terms: HPV vaccines, HPV and oral cancer, HPV and cancer, Gardasil® and prevention of cancer; fields: all; limits: within the last 10 years, humans, English, birth through age 99. The search returned over 5,296 articles. Papers for review were chosen from this list and from the references within selected articles.

Background

HPV is associated with anogenital, skin, and oral and oropharyngeal cancers (**OOPC**)[2-4]. It also is observed in oral squamous cell carcinoma, the most common type of OOPC.[5] Based on epidemiological trends, 53,260 new cases and 10,750 deaths due to OOPC were expected to occur in 2019.[6] HPV is a critical factor, with the HPV 16 strain being the most prevalent.[7] The association between HPV infection and OOPC may be responsible for the recent epidemiologic change with OOPC affecting younger population groups.

Vaccines for prevention of HPV infections via subtypes 16 and 18 have been available since 2006.[8] The Centers for Disease Control and Prevention (**CDC**) found that the prevalence of HPV infection decreased 56 percent among females aged 14-19 years since the vaccine was introduced.[8] A recent study showed 88 percent reduction in prevalence in females and males age 18-33 years.[10] HPV vaccine efficacy against anal and oral infection is high and similar to that against cervical infection.[11] Because the same viral strains are strongly associated with OOPC, it is reasonable to assume that HPV vaccines play an important role in oral pharyngeal cancer prevention. Although there are no studies showing that the HPV vaccine prevents the development of OOPC, it is reasonable to postulate the vaccine's potential since the vaccine has been shown to prevent HPV infection.[9] Despite the increased availability of the HPV vaccines, HPV-related OOPC incidence has continued to increase significantly.[12]

In 2016, the Centers for Disease Control and Prevention (**CDC**) Advisory Committee on Immunization Practices (**ACIP**) recommended a two-dose schedule for children younger than 15 years of age with both doses 6-12 months apart.[13] For children age 15 or older at the time of initial vaccination and for those with immunocompromising conditions, a three-dose series is recommended.[13] The American Academy of Pediatrics (**AAP**) updated their HPV vaccination policy in 2017 to reflect the ACIP/CDC recommendations.[14] Low compliance rates for completion of the vaccination series are due to access, willingness of physicians to discuss with parents, and cost.[14-17]

Adolescent patients have unique needs related to oral healthcare. Anticipatory guidance for adolescent patients includes tobacco and nutritional counseling.[18,19] Given that dental professionals are already involved in secondary and tertiary prevention and, to a limited extent, in the treatment of OOPC, offering primary prevention in dental clinics seems a logical and clinically-appropriate approach. As adolescent patients tend to see the dentist twice yearly and more often than their medical doctor, this is a window of opportunity for the dental professional to provide counseling to the patient and parent about HPV's link to oral cancer and potential benefits of the HPV vaccine.[20]

Policy statement

The AAPD supports measures that prevent OOPC, including the prevention of HPV infection, a critical factor in the development of oral squamous cell carcinoma.

The AAPD encourages oral health care providers to:

- educate patients, parents, and guardians on the serious health consequences of OOPC and the relationship of HPV to OOPC.
- counsel patients, parents, and guardians regarding the HPV vaccination, in accordance with CDC recommendations, as part of anticipatory guidance for adolescent patients.

ABBREVIATIONS
AAP: American Academy of Pediatrics. **AAPD:** American Academy of Pediatric Dentistry. **ACIP:** Advisory Committee on Immunization Practices. **CDC:** Centers for Disease Control and Prevention. **HPV:** Human papilloma virus. **OOPC:** Oral and oropharyngeal cancer.

- routinely examine patients for oral signs of and changes consistent with OOPC.
- follow current literature and consider incorporating other approaches for HPV prevention in their practices so as to minimize the risk of disease transmission.

References

1. American Academy of Pediatric Dentistry. Policy on human papilloma virus vaccinations. Pediatr Dent 2017; 39(6):81-2.
2. National Cancer Institute. HPV and cancer. 2014. Available at: "https://www.cancer.gov/about-cancer/causes-prevention/risk/infectious-agents/hpv-and-cancer". Accessed May 11, 2020.
3. American Cancer Society. Cancer facts and figures 2019. Available at: "https://www.cancer.org/content/dam/cancer-org/research/cancer-facts-and-statistics/annual-cancer-facts-and-figures/2019/cancer-facts-and-figures-2019.pdf". Accessed January 3, 2020.
4. Cogliano V, Baan R, Straif K, Grosse Y, Secretan B, El Ghissassi F. Carcinogenicity of human papillomaviruses. World Health Organization International Agency for Research on Cancer. Lancet Oncol 2005;6:204.
5. Daley E, DeBate R, Dodd V, et al. Exploring awareness, attitudes, and perceived role among oral health providers regarding HPV-related oral cancers. J Public Health Dent 2011;71(2):136-42.
6. American Cancer Society. Cancer A-Z. Oral Cavity and Oropharyngeal Cancer: Causes, Risk Factors, and Prevention. Available at: "https://www.cancer.org/cancer/oral-cavity-and-oropharyngeal-cancer/causes-risks-prevention.html". Accessed May 11, 2020.
7. Weatherspoon DJ, Chattopadhyay A, Boroumand S, Garcia I. Oral cavity and oropharyngeal cancer incidence trends and disparities in the United States: 2000-2010. Cancer Epidemiol 2015;39(4):497-504. Available at: "https://www.ncbi.nlm.nih.gov/pmc/articles/PMC4532587/". Accessed January 3, 2020.
8. Markowitz LE, Dunne EF, Saraiya M, et al. Quadrivalent human papillomavirus vaccine: Recommendations of the Advisory Committee on Immunization Practices (ACIP). MMWR Recomm Rep 2007;56(RR-2):1-24.
9. Markowitz LE, Hariri S, Lin C, et al. Reduction in human papillomavirus (HPV) prevalence among young women following HPV vaccine introduction in the United States, National Health and Nutrition Examination Surveys, 2003-2010. J Infect Dis 2013;208(3):385-93.
10. Chaturvedi AK, Graubard BI, Broutian T, et al. Effect of prophylactic human papillomavirus (HPV) vaccination on oral HPV infections among young adults in the United States. J Clin Oncol 2018;36(3):262-7.
11. Beachler CK. Multisite HPV 16/18 vaccine efficacy against cervical, anal, and oral HPV infection. J Natl Canc Inst 2015;108(1):djv302. Available at: "https://www.ncbi.nlm.nih.gov/pmc/articles/PMC4862406/". Accessed October 31, 2020.
12. Senkomago V, Henley SJ, Thomas CC, Mix JM. Markowitz LE, Saraiya M. Human papillomavirus—Attributable cancers—United States 2012-2015. MMWR Mor Mortal Wkly Rep 2019;68:724-8. Accessed January 3, 2020.
13. Meites E, Kempe A, Markowitz LE. Use of a 2-dose schedule for human papillomavirus vaccination — Updated recommendations of the Advisory Committee on Immunization Practices. Morb Mortal Wkly Rep 2016;65(49):1405-8. Available at: "https://www.cdc.gov/mmwr/volumes/65/wr/mm6549a5.htm". Accessed October 31, 2020.
14. American Academy of Pediatrics. HPV Vaccine Implementation Guidance Updated February 2017. Available at: "https://www.aap.org/en-us/Documents/immunization_hpvimplementationguidance.pdf". Accessed January 3, 2020.
15. McRee AG. HPV vaccine hesitancy: Findings from a statewide survey of health care providers. J Pediatr Health Care 2014;28(6):541-9.
16. Siddiqui M, Salmon DA, Omer SB. Epidemiology of vaccine hesitancy in the United States. Hum Vaccin Immunother 2013;9(12):2643-8.
17. Henrikson NB, Opel DJ, Grothaus L, et al. Physician communication training and parental vaccine hesitancy: A randomized trial. Pediatrics 2015;136(1):70-9.
18. American Academy of Pediatric Dentistry. Adolescent oral health care. The Reference Manual of Pediatric Dentistry. Chicago, Ill.: American Academy of Pediatric Dentistry; 2020:257-66.
19. American Academy of Pediatric Dentistry. Periodicity of examination, preventive dental services, anticipatory guidance/counseling, and oral treatment for infants, children, and adolescents. The Reference Manual of Pediatric Dentistry. Chicago, Ill.: American Academy of Pediatric Dentistry; 2020:232-42.
20. Irwin CE Jr, Adams SH, Park MJ, Newacheck PW. Preventive care for adolescents: Few get visits and fewer get services. Pediatr 2009;123(4):e565-72. Available at: "https://dx.doi.org/10.1542/peds.2008-2601". Accessed January 3, 2020.

I Vaccinate Toolkit

Poster #1:

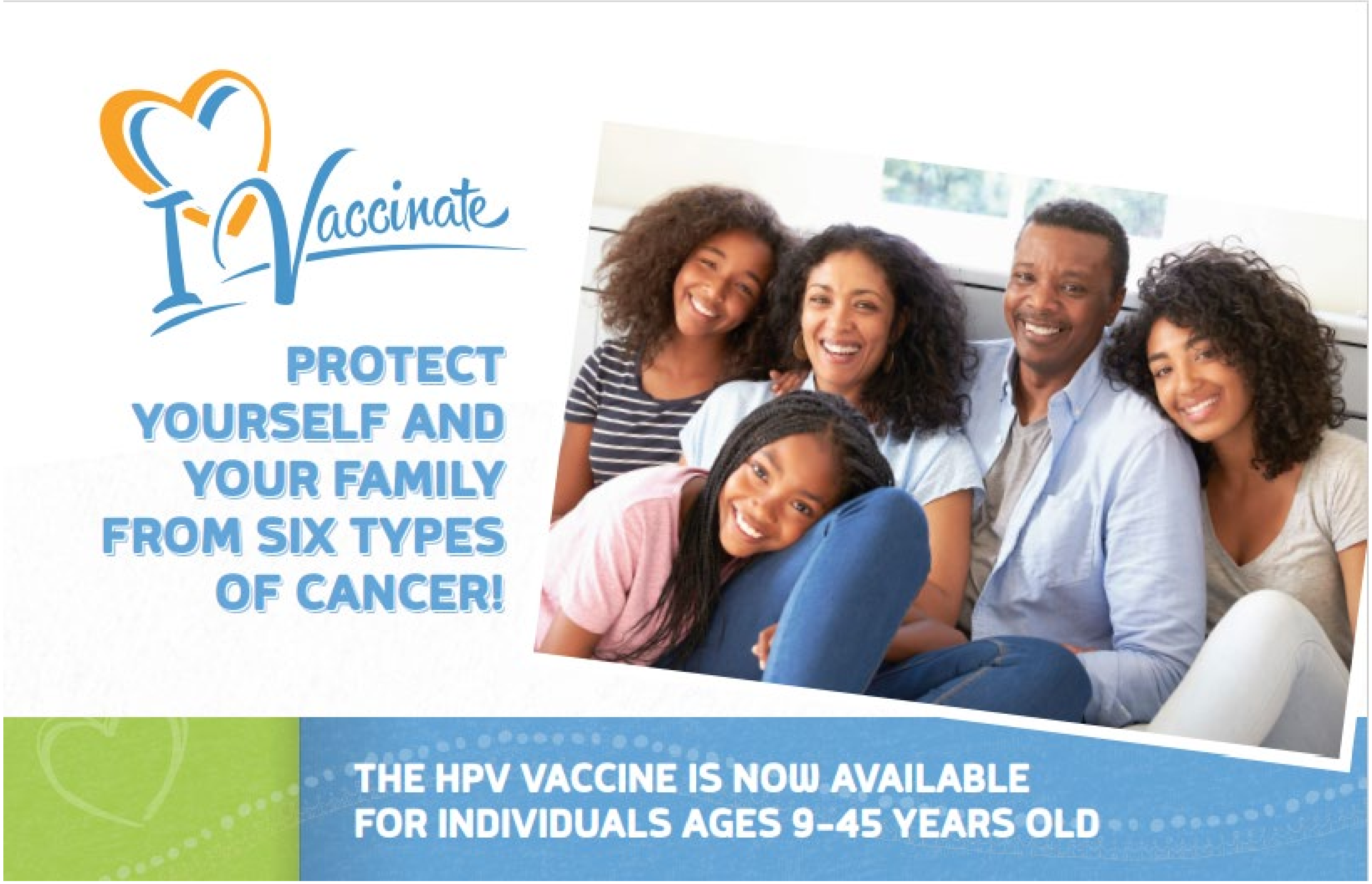

Poster #2:

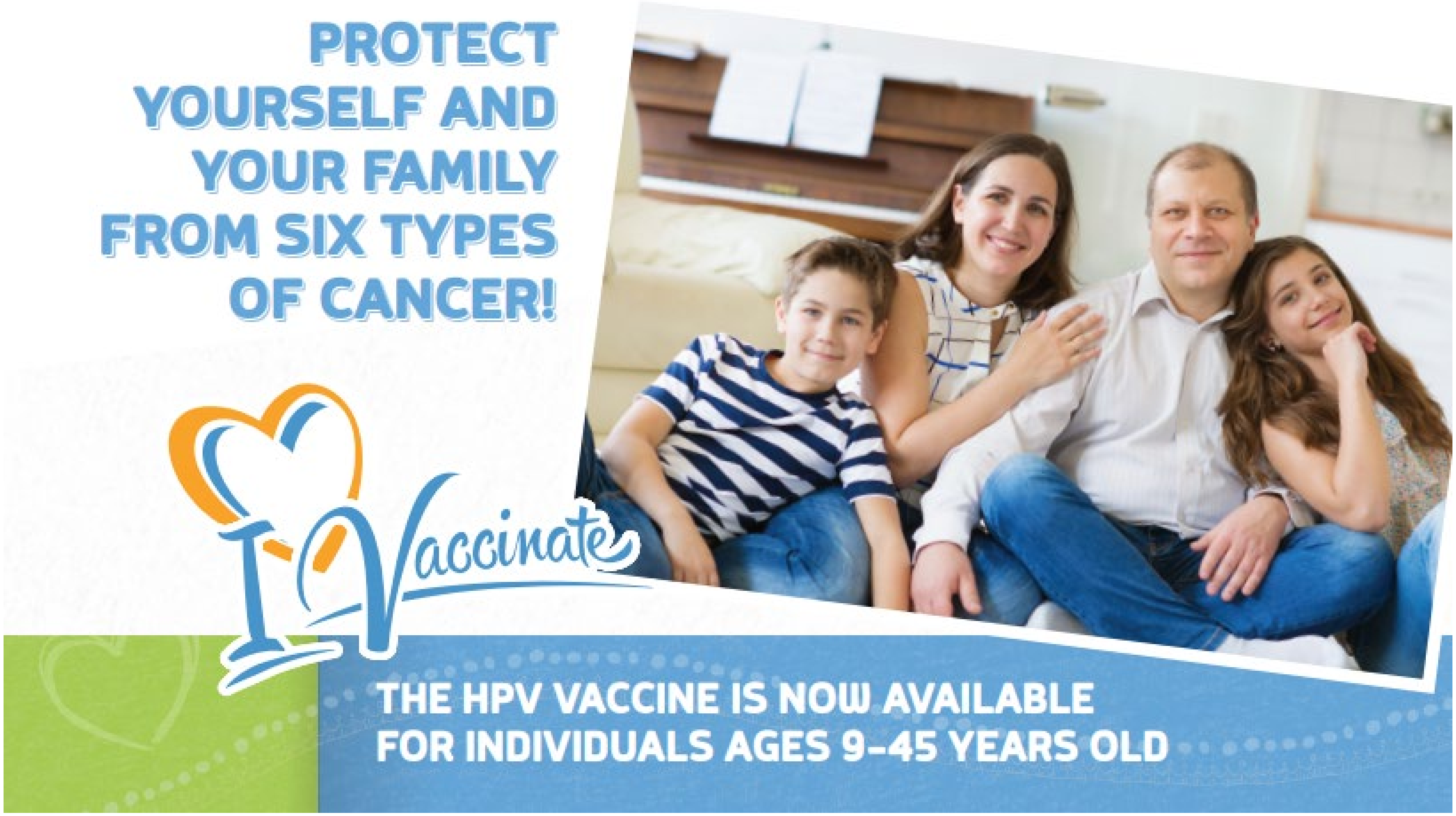
PROTECT YOURSELF AND YOUR FAMILY FROM SIX TYPES OF CANCER!
I Vaccinate
THE HPV VACCINE IS NOW AVAILABLE
FOR INDIVIDUALS AGES 9-45 YEARS OLD

Pamphlet—Pg 1:

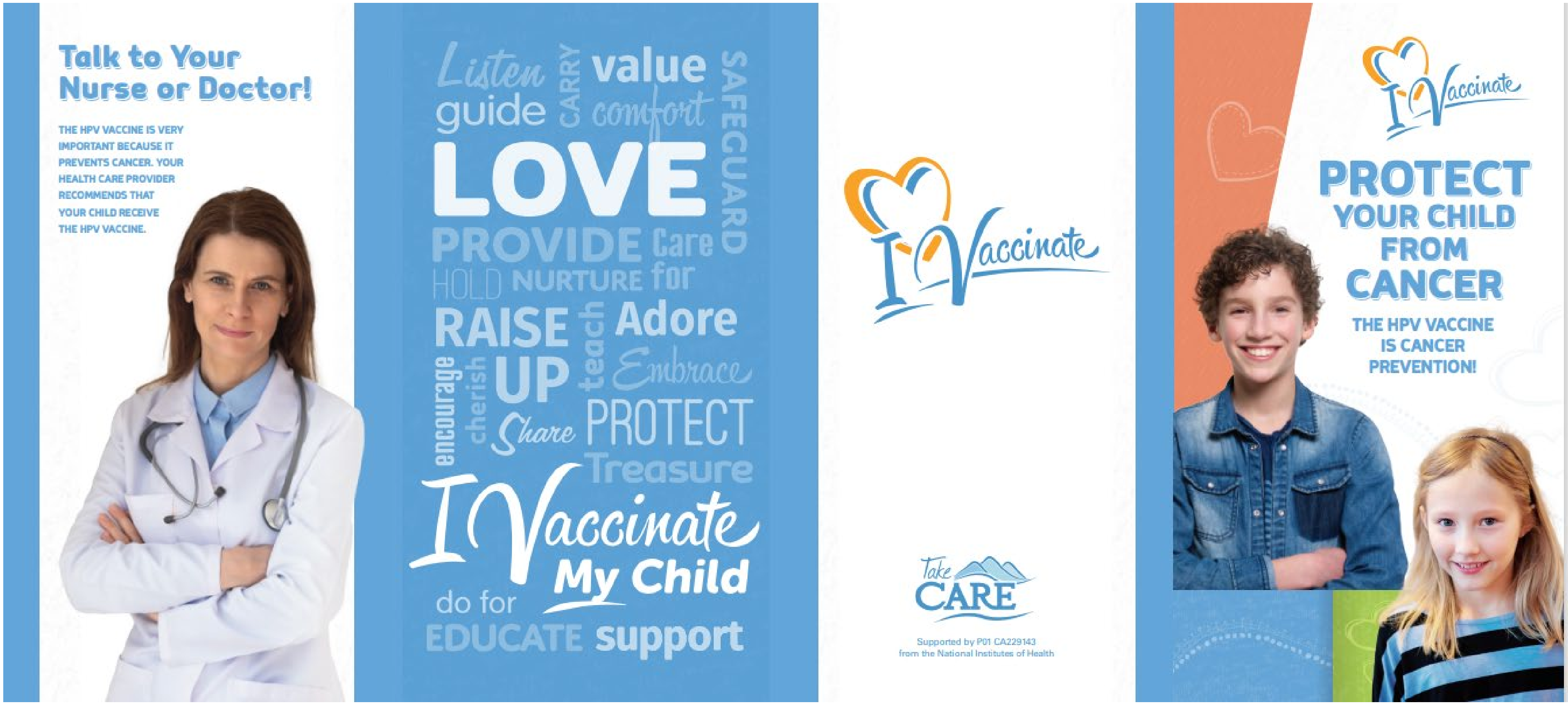

Pamphlet—Pg2

The HPV Vaccine Protects Your Child From Cancer

We Now Have A Chance To Prevent Cancer!

WHAT IS HPV?

HPV is Human Papillomavirus. HPV is a common virus (very small organism) that may infect males and females. Like all viruses it has a few different types called "strains" and they get shared between people. Infection with HPV happens by:

- Sharing behaviors (Sharing food, drink, tobacco products)
- Kissing
- Any type of intimate or sexual contact

HPV can cause cancers of the cervix, vagina and vulva, penis, anus, and the mouth or throat. There are about 26,000 cases of these cancers each year– and most can now be prevented with the HPV vaccine!

Nearly 80 million people in the U.S., 1 in every 4, are infected with at least one strain of HPV.

HPV infection has been found in 46% of females prior to first vaginal sex.
Shew, J Infect Dis. 2013

For More Information:

aap.org
ama-assn.org
asco.org
cancer.gov
cancer.org
cdc.gov/hpv/parents
fda.org
aafp.org/patient-care/public-health/immunizations/disease-population/hpv.html

1 THE HPV VACCINE IS STRONGLY RECOMMENDED BY HEALTHCARE PROFESSIONALS

The experts all agree, including the Advisory Committee on Immunization Practices, American Academy of Pediatrics, American Academy of Family Physicians, World Health Organization, American Society of Clinical Oncology, Center for Disease Control, National Cancer Institute, and the Food and Drug Administration, that the HPV vaccine is safe.

2 HPV VACCINE IS MOST EFFECTIVE GIVEN AT AGE 11-12

CDC recommends that 11 and 12 year-olds receive two doses of HPV vaccine at least six to twelve months apart. It can be given with two other childhood vaccines- TDAP and meningococcal. Ideally, the 2-dose HPV series should be completed by age 13.

Vaccines work before people are infected with a virus. Vaccinating at ages 11 to 12 will give the best protection possible. Studies show children respond much better at that age.

Starting at age 15 and for adults up to age 45, three doses of the HPV vaccine are needed, with the second dose occurring 1-2 months after the first dose and the third dose occurring 6 months after the first dose.

HPV Vaccine for All Genders

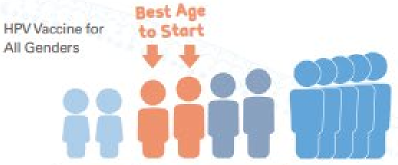

Ages	9	10	11	12	13	14	15 —— 45
Doses	2		2				3

3 HPV VACCINE IS EFFECTIVE, LONG-LASTING, AND SAFE:

EFFECTIVE

Prevalence of 4 HPV types declined in 14-19 year olds by more than half in U.S. after the HPV vaccine was introduced.

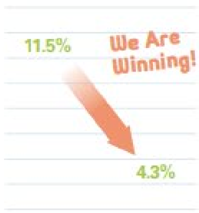

LONG-LASTING

Studies suggest that vaccine protection is long-lasting. There is no evidence of waning immunity over 10 years.

SAFE

Studies found the HPV vaccine is as safe as the TDAP and meningococcal vaccines.

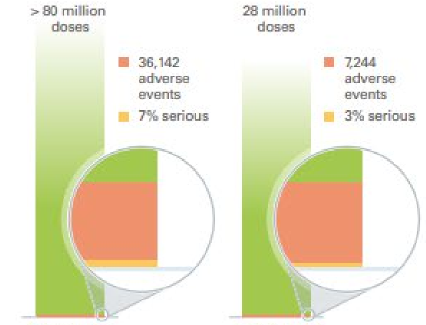

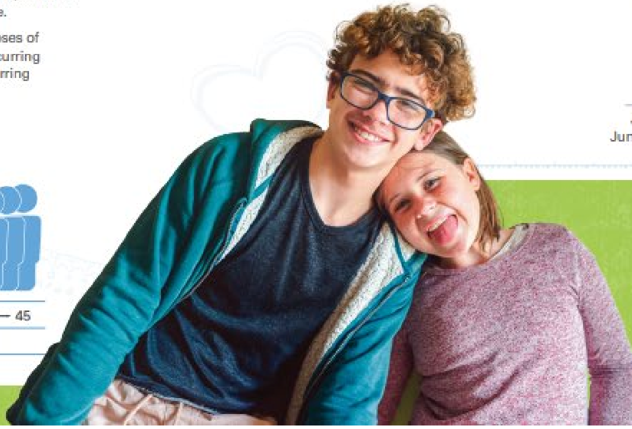

There are stories in the media and online about vaccines that may concern you. However, the HPV vaccine has been carefully studied for many years by medical and scientific experts.

Vaccines, like any medication, can have side effects. With the HPV vaccination this could include pain, swelling, and/or redness where the shot is given, or possibly headache.

Sometimes kids faint when they get shots and they could be injured if they fall. We'll protect your child by having them stay seated after the shot.

Family Fact Sheet—Pg 1:

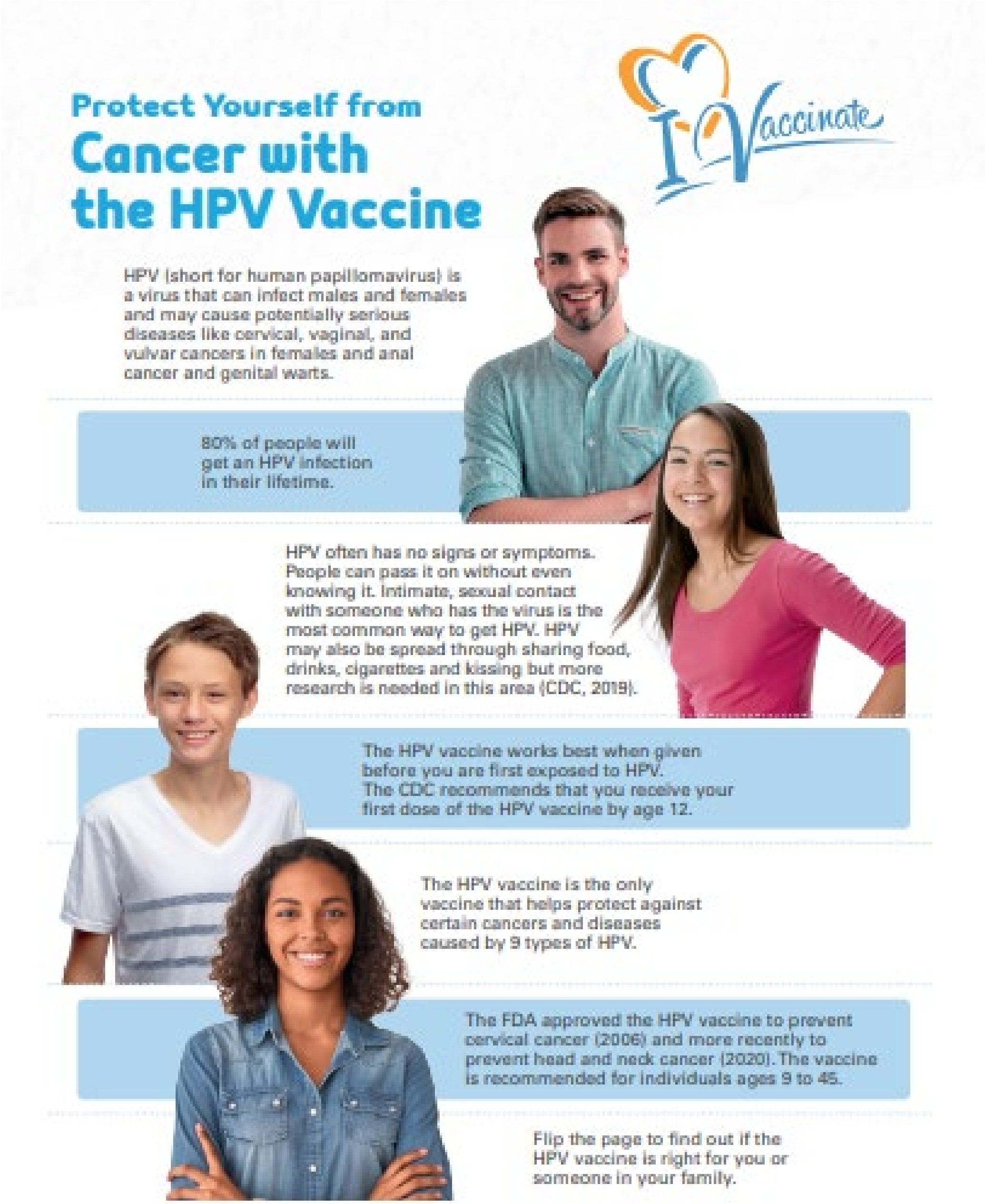

Protect Yourself from Cancer with the HPV Vaccine

I Vaccinate

HPV (short for human papillomavirus) is a virus that can infect males and females and may cause potentially serious diseases like cervical, vaginal, and vulvar cancers in females and anal cancer and genital warts.

80% of people will get an HPV infection in their lifetime.

HPV often has no signs or symptoms. People can pass it on without even knowing it. Intimate, sexual contact with someone who has the virus is the most common way to get HPV. HPV may also be spread through sharing food, drinks, cigarettes and kissing but more research is needed in this area (CDC, 2019).

The HPV vaccine works best when given before you are first exposed to HPV. The CDC recommends that you receive your first dose of the HPV vaccine by age 12.

The HPV vaccine is the only vaccine that helps protect against certain cancers and diseases caused by 9 types of HPV.

The FDA approved the HPV vaccine to prevent cervical cancer (2006) and more recently to prevent head and neck cancer (2020). The vaccine is recommended for individuals ages 9 to 45.

Flip the page to find out if the HPV vaccine is right for you or someone in your family.

Family Fact Sheet—Pg 2:

I Vaccinate

Find out if the HPV vaccine is right for you!

How Old Are You?

< 9	The HPV vaccine is NOT approved for children younger than age 9. (FDA 2014).
9-10	The HPV vaccine is approved to be given as early as age 9 (FDA 2014).
11-12	The HPV vaccine should be given to all boys and girls ages 11 to 12 at their annual well check, along with the TDAP and meningococcal vaccines (CDC 2019).
13-14	It is best to receive the HPV vaccine before age 15 (CDC 2019).
15-26	A catch-up HPV vaccine is highly recommended if you have not already received the HPV vaccine.
27-45	The HPV vaccine is now available for individuals older than age 26 (FDA 2018). Talk to your doctor!
> 45	The FDA has NOT approved the HPV vaccine for persons over the age of 45 (FDA 2018).

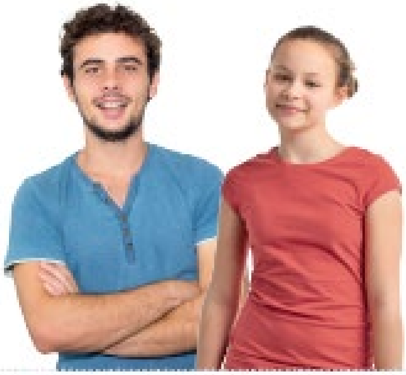

Ages 9-14: You will receive the vaccine in 2 doses, the second dose given 6-12 months after the first.

Ages 15-45: You will receive the vaccine in 3 doses given over 6 months.

Protect Yourself!

Ask your doctor about receiving your first dose of the HPV vaccine today!

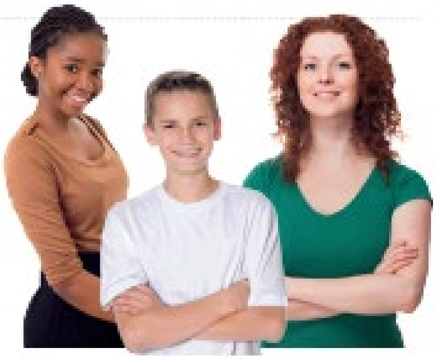

Take CARE

Supported by [illegible] from the National Institutes of Health

Adult Fact Sheet—Pg 1:

It's Not Too Late!

If you are 18 to 45, you can still receive the HPV vaccine and protect yourself

I Vaccinate

What is HPV?

HPV (short for human papillomavirus) is a virus that can infect males and females and may cause potentially serious diseases including six types of cancers and genital warts.

HPV is very common

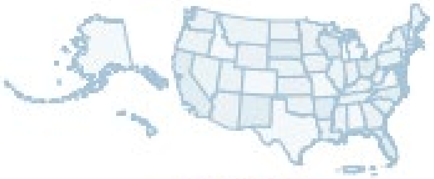

~79 million

people in the United States are currently infected with HPV (CDC, 2019)

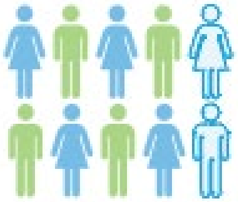

80% of people

will get an HPV infection in their lifetime

What can the HPV vaccine help protect against?

The HPV vaccine is the only vaccine that helps protect against certain cancers and diseases caused by HPV: cervical, vaginal, and vulvar cancers in females, penile in men and anal, oropharyngeal (head and neck) cancers in both men and women.

The FDA approved the HPV vaccine to prevent cervical cancer (2006) and more recently to prevent head and neck cancer (2020).

For most, HPV clears on its own. But, for others, HPV can cause certain cancers and disease. There is no way to predict who will or won't clear the virus.

Today Dose 1 | 2 Months Dose 2 | 6 Months Dose 3

For ages 18-45

The HPV vaccine is a shot that is usually given through the arm muscle and is given as three doses over 6 months.

Adult Fact Sheet—Pg 2:

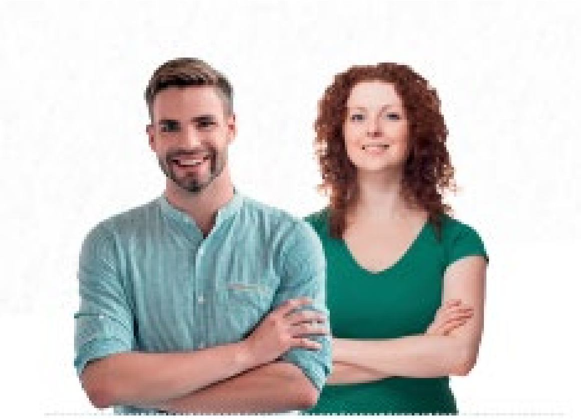

Protect Yourself

Ask your doctor about the HPV vaccine today

HPV is spread through intimate skin-to-skin contact:

Oral Sex Vaginal Sex Anal Sex

HPV often has no symptoms. People can get the virus and pass it on without even knowing it.

The HPV vaccine may not fully protect everyone, nor will it protect against diseases caused by other HPV types or against diseases not caused by HPV. The HPV vaccine does not prevent all types of cervical cancer, so it's important for women to continue routine cervical cancer screenings. The HPV vaccine does not treat cancer or genital warts.

Learn More

GARDASIL9.com/Adults
https://www.cdc.gov/hpv/parents/about-hpv.html
https://www.cdc.gov/vaccines/schedules/hcp/imz/adult.html#table-age
https://www.fda.gov/vaccines-blood-biologics/vaccines/gardasil-9

I Vaccinate

How Old Are You?

18 19 20 21 22 23 24 25 26	The CDC highly recommends a catch-up HPV vaccine if you have not already received the HPV vaccine.
27 28 29 30 31 32 33 34 35 36 37 38 39 40 41 42 43 44 45	Talk to your doctor about your risk for new HPV infections and the benefits of vaccination.
46+	The HPV vaccine is no longer recommended. Talk to your doctor to learn more.

Take CARE

Supported by P01 [illegible] from the National Institutes of Health

Safety Sheet—Pg 1:

I Vaccinate

The HPV Vaccine is

Safe and Effective

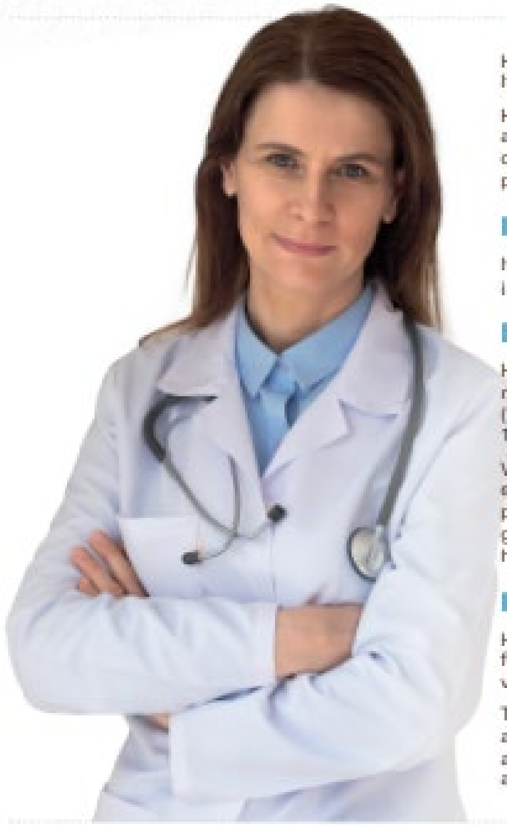

HPV stands for "human papillomavirus." It is a virus that causes cancer.

HPV can be passed from one person to another through intimate activity (including oral sex); sharing food, drinks and tobacco products; and kissing.

It's NOT a New Vaccine!

It was approved for use in the U.S. in females in 2006 and in males in 2009.

It's Safe!

HPV vaccine is just as safe as the meningococcal and tetanus/whopping cough (TDAP) vaccines- the other ones given at the 11-12 year well-check visit.

Vaccines, like any medication, can have side effects. For HPV vaccine, these could include pain, swelling and/or redness where the shot is given. Your child might also complain of a mild headache, nausea, or dizziness.

It Works!

HPV infection was reduced by 86% among females 14 – 19 years of age since the vaccine became available!

The FDA expanded the vaccine to include all individuals ages 9 to 45 in 2018 and approved the vaccine to prevent head and neck cancer in 2020.

We Strongly Recommend the HPV Vaccine to Prevent Six Types of Cancers!

Safety Sheet—Pg 2:

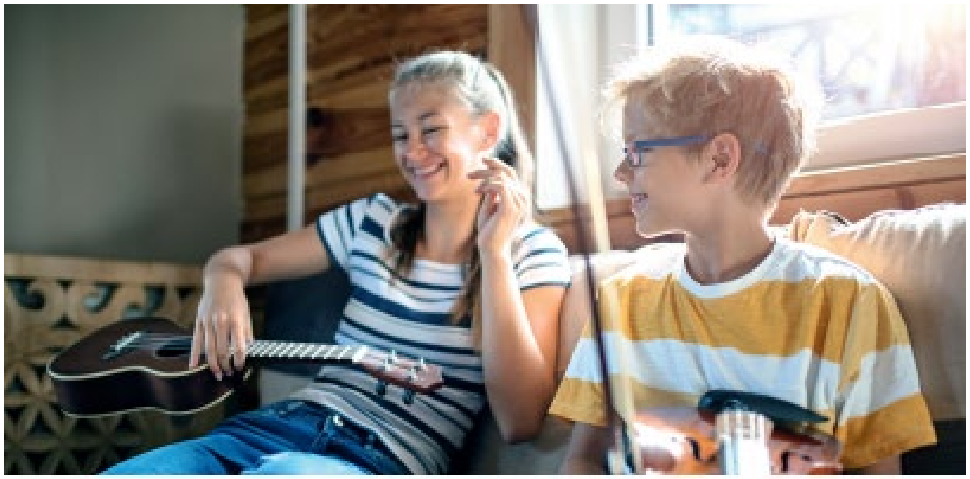

- Vaccines work best when given before someone is first exposed to a virus, such as HPV.
  - This helps guarantee protection (immunity) by the time children are older and more likely to be exposed to the virus.
  - A child's immune system (the part of the body that keeps them healthy) works better than an adult's.
- Your child should be given the first dose of HPV vaccine by age 12.
- The second dose should be given 6 to 12 months after the first.
- Your child should also get two other vaccines at the same time as HPV: meningococcal (for meningitis) and TDAP (for tetanus, diphtheria and pertussis or whooping cough).
- Talk to your child's doctor if your child is older than age 12 and has not had the HPV vaccine. It's not too late to catch up!

The following organizations also strongly recommend the HPV vaccine:

American Academy of Pediatrics

American Academy of Family Physicians

American College of Obstetricians & Gynecologists

Advisory Committee on Immunization Practices

World Health Organization

National Cancer Institute

Centers for Disease Control and Prevention

American Society of Clinical Oncology

Food and Drug Administration

Take CARE

Supported by [illegible] from the National Institutes of Health

Provider talking tips guide ("Reframe Conversation Sheet")—Pg 1:

You Can Help Protect Our Children From HPV-Related Cancers

REFRAME THE CONVERSATION

I ♥ Vaccinate

A healthcare professional's recommendation is the single most important factor in a parent's decision to accept the HPV vaccine for their child.

Research shows* that simply changing the wording used to introduce the HPV vaccine makes a tremendous difference. *"Your child is due for vaccinations today to help protect against meningitis, HPV cancers, and pertussis. We'll give those shots at the end of the visit."*

With this strong and clear message, you convey that HPV vaccine is a normal, recommended vaccine, not a controversial or optional vaccine.

- Recommend the HPV vaccine series the same way you recommend the other adolescent vaccines.
- Parents may be interested in vaccinating, yet still have questions. Some parents might just need additional information from you, the clinician they trust.

Taking the time to answer their questions and address their concerns can help parents accept HPV vaccination.

Providers weaken the HPV vaccination recommendation when*...

- HPV vaccine is presented as 'optional' whereas other adolescent vaccines are recommended.
- Mixed or negative opinions about the 'new vaccine' and concerns over safety/efficacy are expressed by providers to parents.
- Providers are hesitant to engage in discussion when parents expressed reluctance.
- Providers share parents' views that teens are not at risk for HPV and could delay vaccination until older.

Message To Parents:

The HPV vaccine is

1. Strongly recommended by healthcare professionals
2. Most effective if given at age 11-12
3. Safe, effective and long lasting

* Goff S et al. Vaccine 2011; 10:7343-9 Hughes C et al. BMC Pediatrics 2011;11:7

Provider talking tips guide (“Reframe Conversation Sheet”)—Pg 2:

You Can Help Protect Our Children From HPV-Related Cancers

Your help is needed to increase HPV vaccination rates

Nearly 80 million people in the U.S., 1 in every 4, are infected with at least one strain of HPV.

HPV vaccine coverage has stagnated at 50% (up-to-date, 13 -17 year olds) nationally, greatly missing the 80% target necessary to effectively stop the spread of the disease. For each year we stay at current vaccination rates, girls and boys will go on to acquire cervical, oral, anal and other HPV-related cancers.

The HPV vaccination has fallen short of target levels due to:

- Misinformation, negative beliefs and attitudes
- Lack of strong recommendation from health care providers
- Fear of side effects and overall safety

CDC recommendation *Updated October 2019*

Fewer Shots Offer More Incentive to Prevent HPV Cancers

CDC recommends that 11-12-year-olds receive two doses of HPV vaccine six to twelve months apart rather than the previously recommended three doses.

A 2-dose schedule is now approved for children aged 9-14.

- Bundle the HPV vaccine with other age appropriate vaccines at the age 11-12 well-child visit
- Return for 6 to 12 month booster
- Complete series by age 13
- Facilitate "late" vaccination for 13-26 and some 27-45 year olds
- Teens and young adults age 15 and older need 3 doses within 6 months
- Use 9-valent vaccine

FDA approval *Updated June 2020*

The FDA approved an expanded indication for the HPV 9-valent vaccine, recombinant for the prevention of oropharyngeal and other head and neck cancers caused by HPV types 16, 18, 31, 33, 45, 52, and 58.

Methods of HPV Transmission:

Transmission occurs through

- Sharing behaviors (Sharing food, drink, tobacco products)
- Kissing
- Oral sex
- Vaginal sex
- Anal sex

In one study, HPV infection was found in 46% of females prior to first vaginal sex.*

* Shew, J Infect Dis. 2012

Provider talking tips guide (“Reframe Conversation Sheet”)—Pg 3:

Tips to Help You Talk to Parents about the HPV Vaccine:

CDC Research Shows:	Try Saying To Parents:
Disease prevalence is not understood, and parents are unclear about what the vaccine actually protects against.	HPV can cause cancers of the cervix, vagina and vulva in women, cancer of the penis in men and cancers of the anus and the mouth or throat in both women and men. **There are about 35,000 cases of these HPV-related cancers each year– and most could be prevented with the HPV vaccine.** There are also many more precancerous conditions requiring treatment that can have lasting effects.
Parents might believe their child won't be exposed to HPV because they aren't sexually active or may not be for a long time.	**HPV is so common that almost everyone will be infected at some point.** It is estimated that 79 million Americans are currently infected with 14 million new HPV infections each year. Most people infected will never know. So even if your son/daughter waits until marriage to have sex, or only has one partner in the future, he/she could still be exposed if their partner has been exposed.
Parents may be concerned that vaccinating may be perceived by the child as permission to have sex.	Research has shown that getting the HPV vaccine does not make kids more likely to be sexually active or start having sex at a younger age.
The "HPV vaccine is cancer prevention" message resonates strongly with parents. In addition, studies show that a strong recommendation from you is the single best predictor of vaccination.	The HPV vaccine is very important because it prevents cancer. I want your child to be protected from cancer. That's why I'm recommending that your daughter/son receive the first dose of HPV vaccine today.
Parents want a concrete reason to understand the recommendation that 11 or 12 year olds receive the HPV vaccine.	We're vaccinating today so your child will have the best protection possible long before the start of any kind of sexual activity or other transmission exposure. We vaccinate people **before** they are exposed to an infection, as is the case with measles and the other recommended childhood vaccines. Similarly, we want to vaccinate children well before they get exposed to HPV. More simply, we want to have the immunity in place before the exposure happens. Studies show children respond much better.
Would you get HPV vaccine for your kids?	Yes*, I have given the HPV vaccine to my child (or grandchild, etc.) because I believe in the importance of this cancer-preventing vaccine. The American Academy of Pediatrics, the American Academy of Family Physicians, cancer centers, and the CDC also agree that getting the HPV vaccine is very important for your child.<br>*if true
I have some concerns about the safety of the vaccine—I keep reading things online that say HPV vaccination isn't safe. Do you really know if it's safe?	I know there are stories in the media and online about vaccines, and I can see how that could concern you. However, I want you to know that HPV vaccine has been carefully studied for many years by medical and scientific experts. I believe HPV vaccine is very safe. Vaccines, like any medication, can have side effects. With HPV vaccination this could include pain, swelling, and/or redness where the shot is given, or possibly headache. Sometimes kids faint when they get shots and they could be injured if they fall from fainting. We'll protect your child by having them stay seated after the shot.

Provider talking tips guide ("Reframe Conversation Sheet")—Pg 4:

You Can Help Protect Our Children From HPV-Related Cancers

HPV vaccine is:

Safe

Studies found the HPV vaccine as safe as TDAP and meningococcal vaccines.

Effective

Prevalence of HPV types declined by more than half in U.S.

The vaccine is effective for ages 9-26 and people up to 45 years of age.

Long-Lasting

Studies suggest that vaccine protection is long-lasting;
There is no evidence of waning immunity in studies of over 10 years of follow-up.

Facilitate vaccine adherence

Utilize Electronic Medical Records to track and send reminder alerts for recommended vaccinations and boosters

Track vaccination rates to provide feedback

Leverage data analytics to identify characteristics leading to adherence

Recommend the HPV vaccine at time of sports physicals and well-child check-ups

Promoting prevention through the Affordable Care Act

Children & adults are eligible to receive the HPV vaccine recommended by the ACIP (Advisory Committee on Immunization Practices), without any cost-sharing requirements when provided by an in-network provider.*

Next Steps

Give a STRONG recommendation

Ask yourself, how often do you get a chance to prevent cancer?

Start the conversation early and focus it on cancer prevention

Vaccination provides a better antibody response in preteens
and needs to be given well before sexual experimentation begins.

Studies show you should offer a personal story

Share any of the following: own children/grandchildren/close friends' children vaccination, HPV-related cancer case.

Welcome questions from parents, especially about safety

Remind parents that the HPV vaccine is safe and not associated with increased sexual activity.

Garland et al, Prev Med 2011; Ali et al, BMJ 2013
Markowitz JID 2013; Nsouli-Maktabi MSMR 2013

* http://www.hhs.gov/healthcare/facts-and-features/fact-sheets/aca-and-immunization/index.html

Take CARE

Supported by [illegible] from the National Institutes of Health

AAP poster

Answering Questions About HPV Vaccine: A Guide for Dental Professionals

Make a strong recommendation.

Ask parents if their child has completed the Human Papilloma Virus (HPV) vaccine series. Let them know that you strongly support giving the HPV vaccine to children 11 - 12 years of age to protect them from HPV-associated cancers.

Be ready to accurately answer parents' questions.

Usually, letting parents know that HPV causes oropharyngeal cancer (a form of throat cancer) is enough. If a parent needs more information be ready to address their questions and help them understand why you recommend the HPV vaccine. Suggest families contact their pediatrician for more information and access facts about the HPV vaccine from the Centers for Disease Control and Prevention (CDC) to help you with the conversation: https://www.cdc.gov/vaccines/vpd/hpv/public/index.html

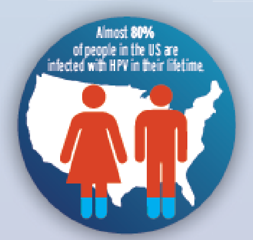

Almost everyone will be infected with HPV at some point in their lives.

Below are some things that parents might say and tips on how to respond.

HPV VACCINE IS IMPORTANT

Is my child at risk for HPV?

- Almost everyone will be infected at some time in their lives. Your pediatrician can help protect your child from the cancers caused by the virus by completing the HPV vaccine series before age 13.

Why is HPV vaccination recommended at ages 11 or 12?

- The immune system of a young adolescent responds better than an older adolescent.

Would you give HPV vaccine to your kids?

- Protecting children, including my own, from HPV is very important. This vaccine is able to protect them from HPV-related cancers.

HPV VACCINE IS EFFECTIVE

How do you know if the vaccine works?

- Ongoing studies show that HPV vaccination works very well. Since becoming available in 2006, this vaccine already has decreased HPV infection, genital warts, and precancers of the cervix in young people.

Why do boys need HPV vaccine?

- In men, HPV infection can cause cancers of the throat as well as the penis and anus. By vaccinating your son as recommended, he will be better protected against the HPV types that can cause these diseases.

HPV VACCINE IS SAFE

Is HPV vaccination safe?

- The HPV vaccine is very safe. More than 80 million doses have been given in the US and it has been studied for more than ten years by medical and scientific experts. This vaccine is able to protect them from HPV-related cancers.

What are the possible vaccine side effects?

- HPV vaccine, like any vaccine, can cause injection site pain, swelling, or redness. Some adolescents may have a headache or even faint after they get shots. Fainting may lead to injury if the child falls, so offices typically have patients stay seated for a while after receiving the shot.

By ensuring that patients 11 or 12 years old get the HPV vaccine, we can give them a chance at a future free from HPV-associated cancers.

HPV YOU ARE THE KEY TO CANCER PREVENTION

American Academy of Pediatrics
DEDICATED TO THE HEALTH OF ALL CHILDREN®

For More Information: aap.org/oralhealth • aap.org/hpvtoolkit
email: HPV@aap.org

This factsheet is supported by the Grant or Cooperative Agreement Number, 6NH23IP000952-04-01, funded by the Centers for Disease Control and Prevention. Its contents are solely the responsibility of the authors and do not necessarily represent the official views of the Centers for Disease Control and Prevention or the Department of Health and Human Services.

The recommendations in this publication do not indicate an exclusive course of treatment or serve as a standard of medical care. Variations, taking into account individual circumstances, may be appropriate. This fact sheet has been developed by the American Academy of Pediatrics. The authors, editors, and contributors are expert authorities in the field of pediatrics. No commercial involvement of any kind has been solicited or accepted in the development of the content of this publication.

Copyright © 2018 American Academy of Pediatrics. You may download or print from our website for personal reference only. To reproduce in any form for commercial purposes, please contact the American Academy of Pediatrics.
