# Supplementary material for: STEPS (Study To Examine Parent, Patient/Dental Provider Systems) to Prevent Human Papillomavirus (HPV)-Related Cancers: A Piloted Dental Patient and Provider Evaluation of Current and Future HPV Education
Source: J Cancer Educ. 2024 Jul 4;40(1):44–53. doi: 10.1007/s13187-024-02465-2 (PMC11846729; doi:10.1007/s13187-024-02465-2)
Supplement: Supplementary file 3 — Supplementary Material 3 [file 13187_2024_2465_MOESM3_ESM.pdf]

Article Title: STEPS To Prevent Human Papillomavirus (HPV)-related Cancers: A Piloted Dental Patient and Provider Evaluation of Current and Future HPV Education

Journal Name: Journal of Cancer Education

Author Names: Kelsey H. Jordan; Julie A. Stephens; Kaleigh Niles; Nina Hoffmeyer; Michael L. Pennell; Jill M. Oliveri; Electra D. Paskett

Corresponding Author: Kelsey H. Jordan

Affiliation: Division of Population Sciences, Comprehensive Cancer Center, The Ohio State University, Columbus, Ohio, USA

Email Address: kelsey.jordan@osumc.edu

Description of Existing HPV Educational Materials for Focus Group & Independent Survey Review

Videos

Parent and adult focus group participants watched four videos while provider focus group participants watched a total of seven videos (i.e., four from the parent/adult focus groups and an additional three geared toward health care providers (i.e., dentists and pediatricians)). Videos ranged in length from 40 seconds to nearly three minutes.

Parent/Adult Video 1 ("Are You HUMAN?" by Team Maureen)[1]: This is a one-minute animated video that explains what HPV is, its links to cancer and some kinds of warts, that vaccination can prevent HPV, and who should get vaccinated. The video ends with a recommendation to visit your doctor and dentist every year and complete Pap/HPV and oral cancer screening as advised. This video is also Provider Video 4. https://www.youtube.com/watch?v=1Dm2DtUy0fM&feature=emb_imp_woyt

Parent/Adult Video 2 ("HPV Campaign - Mission HPV Cancer Free" by the American Cancer Society, ACS)[2]: This 1 ½-minute video points out that the vaccine is based on science, is not new, and is safe. It links HPV to six different types of cancer and invites the viewer to be part of the success story in eliminating HPV cancers. The narrative notes ACS's goal of 80% vaccinated by 2026. This video is also Provider Video 5. https://www.youtube.com/watch?v=S_nNo_vhMsk&feature=emb_imp_woyt

Parent/Adult Video 3 ("HPV Vaccination Animation - What's Best for Our Kids?" by Louisiana State University, LSU)[3]: This is a 40-second animated video that leads with a common goal of doing what is best for our kids in our desire to protect them. It points out the link between HPV and throat cancers, with men having a higher prevalence of this type of cancer. The video then mentions the HPV vaccine as a prevention tool, noting its safety and effectiveness. Sadly, fewer boys (compared to girls) get vaccinated. For more information, the video refers viewers to www.cancer.org. This video is also Provider Video 6. https://www.youtube.com/watch?v=CEM7AnAWknI&feature=emb_imp_woyt

Parent/Adult Video 4 ("Steve's Story – Oral Cancer Caused by HPV" by the Minnesota Department of Health, MN Health)[4]: This nearly three-minute video tells the story of a young oral cancer survivor (male age 28) and features him, his newlywed wife, and his physician. It links head and neck cancers to HPV infection and makes the case that HPV vaccination in children can prevent head and neck cancer in adults. The video concludes with a recommendation to prevent cancer before it ever starts and to talk with your health care provider about getting the HPV vaccine. This video is also Provider Video 7. https://www.youtube.com/watch?v=46-li108faU&feature=emb_imp_woyt

Provider Video 1 ("Talking about HPV Vaccination" by ACS)[5]: This 2 ½ minute video is similar in style to Adult/Parent Video 2. It provides talking points for dentists for how to recommend HPV vaccination to their patients: safe; cancer prevention for both boys and girls; age 11 - 12; use "I" statements to make a connection; share information and refer to ACS public website for more information; suggestion to ask pediatrician or nurse about HPV vaccination; ok to say "I don't know the answer to that question, but it's a great question for your child's doctor"; if tense, acknowledge common goal (protect our kids). https://www.youtube.com/watch?v=Eza6MMLxT1g&feature=emb_imp_woyt

Provider Video 2 ("Dr. Casler - Improving HPV Vaccine Recommendation" by the Centers for Disease Control and Prevention, CDC)[6]: This 1 ½-minute video features a female pediatrician and mother and focuses on providers who want to improve HPV vaccine recommendation. She notes that messages to patients and their parents should be simple (infection is common, can cause cancer, & can be prevented) and providers should only answer questions if they are asked. The video also says providers should practice giving a recommendation until they are comfortable. Dr. Casler advises that to patients, providers

need to give a clear bundled recommendation, ask if there are questions, and answer only those questions. https://www.youtube.com/watch?v=VbzgMkDAKsk&feature=emb_imp_woyt

Provider Video 3 ("Dr. Wolynn - Practice-wide HPV Vaccination Recommendation" by the CDC)[7]: This is a 40-second video featuring a male pediatrician and father who describes how his practice routinely recommends HPV cancer prevention. He ensures that all staff are on board with HPV vaccination as cancer prevention to create a shared office culture. https://www.youtube.com/watch?v=nIwbt3F5k-c&feature=emb_imp_woyt

Print Materials

Team Maureen[8]

Up to five print material pieces were reviewed, depending on participant review group: a tri-fold pamphlet, a poster (11 inches x 17 inches), talking tips, a vaccine reminder card, and a policy statement. All materials were produced by the Massachusetts HPV Coalition. The topics featured in each are noted below.

- Tri-fold pamphlet ("Preventing Cancer at the Dentist"): The educational piece covers: (1) What HPV is and its link to cancer; (2) HPV vaccine information; (3) oral, head and neck self-exam guide; (4) prevent cancer checklist (1st box is Get Vaccinated! with a checkmark); (5) HPV vaccine is for everyone.
- Poster (11 x 17 inches): It reiterates pamphlet topics and details in a larger format.
- Talking tips (parent, 18-26 yrs, 27+ yrs): Question (patients may ask) and answer (potential provider responses) style documents to guide dentists through conversations with parents of young patients and adult dental patients, respectively, about the HPV vaccine. Vaccine topics for parents include: importance, eligibility (age, sex), safety & effectiveness, and cost. Adult topics include: disease description, eligibility (ages, HPV status), HPV risk post-vaccination, oral HPV testing, HPV vaccine safety.
- Vaccine reminder card (4 to a page): The reminder card reads: "[Name] is due for the cancer-preventing HPV Vaccine. Call their doctor to schedule an appointment." The Massachusetts HPV Coalition logo is also included.
- HPV vaccine policy statement: Written from the perspective of the American Academy of Pediatric Dentistry, it discusses the purpose and methods for developing a policy statement on HPV vaccines. The document provides background information on the epidemiology of HPV (e.g., rates/trends, HPV strains) and HPV vaccine specifics (e.g., history, efficacy, schedule and dosing, primary prevention dental efforts). It ends by stating the two-part policy statement of encouraging dental providers to 1) educate on HPV and oral and oropharyngeal cancers; and 2) advise patients/guardians on HPV vaccination.

I Vaccinate

Up to six print material pieces were reviewed: a poster (two versions), a pamphlet, a family fact sheet, an adult fact sheet, a safety sheet, and a provider talking guide. All materials were created and produced for a National Cancer Institute (NCI)-funded research initiative (Take CARE).

- Family Posters: There were two versions of family depictions: one Black family – 2 middle aged parents + three tween/teen daughters; one White family – 2 middle aged parents + one tween/teen

daughter & one son age 9 – 11. The message was the same—" Protect yourself and your family from six types of cancer! HPV vaccine is available for people age 9 – 45."

- Pamphlet: The quadri-fold pamphlet featured four white children (2 boys and 2 girls) and a white female provider. Highlights of content included: Vaccine is cancer prevention; strongly recommended by healthcare professionals; most effective when given at age 11 – 12; effective, long-lasting and safe. It also features easy-to-read graphics, a list of additional resources, the recommendation to talk with a nurse or doctor about the HPV vaccine, and a word cloud for sentiments about children.
- Family Fact Sheet: It is a two-page informational handout with the first page featuring seven HPV facts and emphasizing that the HPV vaccine is cancer prevention. The 2nd page features seven age groups (i.e., < 9 through > 45) and the HPV vaccine recommendation for each. The document includes racially diverse photos of children and young adults.
- Adult fact sheet: This resource is a two-page document for 18-45 year olds reviewing what HPV is, the prevalence of HPV, HPV vaccine protection, HPV vaccine eligible ages, and dosing of HPV Vaccine on page 1. Page 2 includes information on HPV transmission routes, additional HPV vaccine protection/guidance, links to additional resources, and an age range graphic indicating appropriate age-related approaches for navigating the HPV vaccination process.
- Safety Sheet: The two-page safety handout features a white female provider on the front and two white children (teenage girl and tween boy) on the back. The front page highlights that the HPV vaccine is not new, it is safe, and it is effective. It includes a printed strong recommendation to vaccinate to prevent six types of cancer at the bottom of the front page. The back page features a list of seven important details about the HPV vaccine, including simultaneous vaccination with the meningococcal and TDAP vaccines and an opportunity to catch up on vaccinations for kids older than age 12. The back also includes a list of organizations that strongly recommend HPV vaccination (e.g., American Academy of Pediatrics, National Cancer Institute, CDC).
- Provider talking tips guide—"Reframe the conversation" (*providers only): A four-page resource for providers to use to facilitate HPV vaccine conversations and recommendations with patients. Page 1 provides details on what to (not) do to strengthen the HPV vaccine recommendation. Page 2 reviews facts about HPV (e.g., prevalence, transmission routes) and the HPV vaccine (e.g., current HPV vaccine rates, CDC HPV vaccine recommendations, FDA vaccine approval). Page 3 provides tips to aid providers in talking to parents about the HPV vaccine, including how to take CDC research findings and translate them into meaningful information for parents. Page 4 is a summary of the main points of the document and lists actionable items for providers to complete to improve HPV vaccination rates, including to facilitate vaccine adherence.

AAP poster[9]

A one-page infographic prepared by the American Academy of Pediatrics for dental providers to use as a guide to answer parents' questions about the HPV vaccine. It covers topics regarding how to make a strong recommendation as well as vaccine importance, safety, and effectiveness.

References

1. *Are you HUMAN?* TeamMaureen. Accessed April 20, 2022. https://www.youtube.com/watch?v=1Dm2DtUy0fM&feature=emb_imp_woyt
2. *HPV Campaign - Mission HPV Cancer Free by the American Cancer Society*. ACS. Accessed April 20, 2022. https://www.youtube.com/watch?v=S_nNo_vhMsk&feature=emb_imp_woyt
3. *HPV Vaccination Animation - What's Best for Our Kids?* LSU. Accessed April 20, 2022. https://www.youtube.com/watch?v=CEM7AnAWknI&feature=emb_imp_woyt
4. *Steve's Story - Oral Cancer Caused by HPV*. MNHealth. Accessed April 20, 2022. https://www.youtube.com/watch?v=46-li108faU&feature=emb_imp_woyt
5. *Talking About HPV Vaccination Video*. ACS. Accessed April 20, 2022. https://www.youtube.com/watch?v=Eza6MMLxT1g&feature=emb_imp_woyt
6. *Improving HPV Vaccine Recommendation--Suggestions from Dr. Alix Casler*. CDC. Accessed April 20, 2022. https://www.youtube.com/watch?v=VbzgMkDAKsk&feature=emb_imp_woyt
7. *Dr. Wolynn Discusses How His Office Routinely Recommends HPV Vaccine*. CDC. Accessed April 20, 2022. https://www.youtube.com/watch?v=nIwbt3F5k-c&feature=emb_imp_woyt
8. Shukla A, Nyambose J, Vanucci R, et al. Evaluating the Effectiveness of Human Papillomavirus Educational Intervention among Oral Health Professionals. *Journal of cancer education : the official journal of the American Association for Cancer Education*. Jul 13 2018;doi:10.1007/s13187-018-1391-z
9. Answering Questions About HPV Vaccine: A Guide for Dental Professionals. 2018. Accessed April 20, 2022. https://hpvroundtable.org/resource-library/
