# Supplementary material for: STEPS (Study To Examine Parent, Patient/Dental Provider Systems) to Prevent Human Papillomavirus (HPV)-Related Cancers: A Piloted Dental Patient and Provider Evaluation of Current and Future HPV Education
Source: J Cancer Educ. 2024 Jul 4;40(1):44–53. doi: 10.1007/s13187-024-02465-2 (PMC11846729; doi:10.1007/s13187-024-02465-2)
Supplement: Supplementary file 4 — Supplementary Material 4 [file 13187_2024_2465_MOESM4_ESM.pdf]

Article Title: STEPS To Prevent Human Papillomavirus (HPV)-related Cancers: A Piloted Dental Patient and Provider Evaluation of Current and Future HPV Education

Journal Name: Journal of Cancer Education

Author Names: Kelsey H. Jordan; Julie A. Stephens; Kaleigh Niles; Nina Hoffmeyer; Michael L. Pennell; Jill M. Oliveri; Electra D. Paskett

Corresponding Author: Kelsey H. Jordan

Affiliation: Division of Population Sciences, Comprehensive Cancer Center, The Ohio State University, Columbus, Ohio, USA

Email Address: kelsey.jordan@osumc.edu

Post-survey HPV Vaccine Focus Group Discussion Guide: Parents

Questions for Review of Educational Materials
VIDEO
Overall
Did the video keep you interested throughout? [**PROBE**: flow (i.e., smoothness, logic), pace, tone]

Was there anything in the video that was difficult for you to comprehend?

Did the video contain anything that was offensive to you? Dislikes?

Audio
Could you understand what the people were actually saying?

Were the words appropriate or were they difficult to understand?

Visual
Were the pictures, titles, and graphics sharp, in focus, and helpful?

Content
Was the purpose/message of the video?

Did you feel the information provided in the video was credible? [**PROBE**: dental office appropriateness, controversial topics, missing information]

Does the video seem to address readily heard concerns and/or barriers to HPV vaccination for parents?

After watching the video, do you think it's important for boys and girls who live in Ohio to get the HPV vaccine? [**PROBE**: advice for debating parents, outstanding vaccine concerns, missing information, extraneous information to remove]

Was it helpful to include the part on the video that focused on how you should ask your child's dentist about the HPV vaccine? Vice versa? [**PROBE**: comfort level for discussions]

PRINT MATERIALS
Overall Appearance
What are your initial impressions? [**PROBE**: thoughts, material type preference]

Do you like the color, font, pictures on these materials? Improvements for material appearance?

Are any of these materials offensive to you? Dislikes?

Content

What is the main message that you get from these materials? [**PROBE**: confusion, personable, language levels]

Information concise yet informative? [**PROBE**: missing content, format, preferred changes]

Do printed materials seem to address readily heard concerns and/or barriers to HPV vaccination for parents?

Do you think these posters are ok to hang in dental practices' waiting/examination rooms?

Are communication tips useful? [**PROBE**: comfort level for discussions]

Would you use these materials to learn more about HPV and its related topics? [**PROBE**: relatedness, appropriateness, impact level]

After reviewing these materials, have your viewpoints on HPV vaccine promotion changed? If so, how?

Post-survey HPV Vaccine Focus Group Discussion Guide: Adult Dental Patients

Questions for Review of Educational Materials

VIDEO

Overall

Did the video keep your interest throughout? [**PROBE**: flow (i.e., smoothness, logic), pace, tone]

Was there anything in the video that was difficult for you to comprehend?

Did the video contain anything that was offensive to you? Dislikes?

Audio

Could you understand what the people were actually saying?

Were the words appropriate or were they difficult to understand?

Visual

Were the pictures, titles, and graphics sharp, in focus, and helpful?

Content

What was the purpose/message of the video?

Did you feel the information provided in the video was credible? [**PROBE**: dental office appropriateness, controversial topics, missing information]

Does the video seem to address readily heard concerns and/or barriers to HPV vaccination for adults?

After watching the video, do you think it's important for adults aged 18 – 45 years old who live in Ohio to get the HPV vaccine? [**PROBE**: advice for debating adults, outstanding vaccine concerns, missing information, extraneous information to remove]

Was it helpful to include the part on the video that focused on how you should ask your dentist about the HPV vaccine? Vice versa? [**PROBE**: comfort level for discussions]

PRINT MATERIALS

Overall Appearance

What are your initial impressions? [**PROBE**: thoughts, material type preference]

Do you like the color, font, pictures on these materials? Improvements for material appearance?

Are any of these materials offensive to you? Dislikes?

Content

What is the main message that you get from these materials? [**PROBE**: confusion, personable, language levels]

Information concise yet informative? [**PROBE**: missing content, format, preferred changes]

Do printed materials seem to address readily heard concerns and/or barriers to HPV vaccination for adults?

Are the communication tips useful? [**PROBE**: comfort level for discussions]

Do you think the posters are ok to hang in dental practices' waiting and examination rooms?

Would you use these materials to learn more about HPV and its related topics? [**PROBE**: relatedness, appropriateness, impact level]

After reviewing these materials, have your viewpoints on HPV vaccine promotion changed? If so, how?

Post-survey HPV Vaccine Focus Group Discussion Guide: Dental Providers

Questions for Review of Provider/Practice Educational Materials
VIDEO
Overall
Did the video keep you interested throughout? [**PROBE**: flow (i.e., smoothness, logic), pace, tone]

Was there anything in the video that was difficult for you to comprehend?

Did the video contain anything that was offensive to you? Dislikes?

Audio
Could you understand what the people were actually saying?

Were words appropriate or were they difficult to understand?

Visual
Were pictures, titles, and graphics sharp, in focus, and helpful?

Content
What was the video's purpose/message?

Did you feel the information provided in the video was credible? [**PROBE**: dental office appropriateness, controversial topics, missing information]

Does the video seem to address readily heard concerns and/or barriers to HPV vaccination?

After watching the video, do you think it's important for people 9 – 45 years old who live in Ohio to get the HPV vaccine? [**PROBE**: advice for debating parents/patients, outstanding vaccine concerns, missing information, extraneous information to remove]

Was it helpful to hear about how patients/parents should ask dental providers about the HPV vaccine? Vice versa? Do you think patients/parents could start a conversation with dental practice staff members about the HPV vaccine? [**PROBE**: comfort level for discussions]

PRINT MATERIALS
Overall Appearance
What are your initial impressions? [**PROBE**: thoughts, material type preference]

Do you like the color, font, pictures on these materials? Improvements for material appearance?

Are any of these materials offensive to you? Dislikes?

Content

What is the main message that you get from these materials? [**PROBE**: confusion, personable, language levels]

Information concise yet informative? [**PROBE**: missing content, format, preferred changes]

Do printed materials seem to address readily heard concerns for/barriers to HPV vaccination for parents? Older patients?

Do you think posters/flyers are ok to hang in dental practice waiting/examination rooms?

Are communication tips useful? [**PROBE**: comfort level for discussions]

Would you and/or your patients likely use these materials to learn more about HPV and its related topics? [PROBE: relatedness, appropriateness, impact level]

After reviewing these materials, have your viewpoints on HPV vaccine promotion changed? If so, how?
