# Supplementary material for: STEPS (Study To Examine Parent, Patient/Dental Provider Systems) to Prevent Human Papillomavirus (HPV)-Related Cancers: A Piloted Dental Patient and Provider Evaluation of Current and Future HPV Education
Source: J Cancer Educ. 2024 Jul 4;40(1):44–53. doi: 10.1007/s13187-024-02465-2 (PMC11846729; doi:10.1007/s13187-024-02465-2)
Supplement: Supplementary file 5 — Supplementary Material 5 [file 13187_2024_2465_MOESM5_ESM.pdf]

Article Title: STEPS To Prevent Human Papillomavirus (HPV)-related Cancers: A Piloted Dental Patient and Provider Evaluation of Current and Future HPV Education

Journal Name: Journal of Cancer Education

Author Names: Kelsey H. Jordan; Julie A. Stephens; Kaleigh Niles; Nina Hoffmeyer; Michael L. Pennell; Jill M. Oliveri; Electra D. Paskett

Corresponding Author: Kelsey H. Jordan

Affiliation: Division of Population Sciences, Comprehensive Cancer Center, The Ohio State University, Columbus, Ohio, USA

Email Address: kelsey.jordan@osumc.edu

HPV Education Material Review by Email Survey: Dental Patients

VIDEO:

Overall

a. Overall, the video was interesting.
Strongly disagree Disagree Neither agree nor disagree Agree Strongly agree
○ Prefer not to answer

b. I understood what the video was trying to teach me.
Strongly disagree Disagree Neither agree nor disagree Agree Strongly agree
○ Prefer not to answer

c. Nothing in the video was offensive to me.
Strongly disagree Disagree Neither agree nor disagree Agree Strongly agree
○ Prefer not to answer

d. Additional comments about items you agreed/disagreed with about the video overall:
____

Audio

e. The speakers were easy to hear.
Strongly disagree Disagree Neither agree nor disagree Agree Strongly agree
○ Prefer not to answer

f. The video used words I could understand.
Strongly disagree Disagree Neither agree nor disagree Agree Strongly agree
○ Prefer not to answer

g. Additional comments about items you agreed/disagreed with about the video audio:
____

Visual

h. Pictures, titles, and/or graphics were helpful.
Strongly disagree Disagree Neither agree nor disagree Agree Strongly agree
○ Prefer not to answer

i. Additional comments about items you agreed/disagreed with about the video visuals:
____

Content

j. The video included information from a trusted source.
Strongly disagree Disagree Neither agree nor disagree Agree Strongly agree

○ Prefer not to answer

k. The video addresses common concerns about the HPV vaccine.
Strongly disagree Disagree Neither agree nor disagree Agree Strongly agree

○ Prefer not to answer

l. After watching the video, I think it is important for people 9-45 years old who live in Ohio to get the HPV vaccine.
Strongly disagree Disagree Neither agree nor disagree Agree Strongly agree

○ Prefer not to answer

m. After watching the video, I would feel comfortable talking to my dental provider about the HPV vaccine.
Strongly disagree Disagree Neither agree nor disagree Agree Strongly agree

○ Prefer not to answer

n. Additional comments about items you agreed/disagreed with about the video content:
____

Paper Materials

Overall Appearance

a. The colors, font, and/or pictures are appropriate.
Strongly disagree Disagree Neither agree nor disagree Agree Strongly agree

○ Prefer not to answer

b. None of the materials offend me.
Strongly disagree Disagree Neither agree nor disagree Agree Strongly agree

○ Prefer not to answer

c. The way the text is formatted makes it easy to read.
Strongly disagree Disagree Neither agree nor disagree Agree Strongly agree

○ Prefer not to answer

d. additional comments about items you agreed/disagreed with about the toolkit's overall appearance: ____

Content

e. The messages in the materials are useful.
Strongly disagree Disagree Neither agree nor disagree Agree Strongly agree

○ Prefer not to answer

f. The printed materials address common concerns about the HPV vaccine.
Strongly disagree Disagree Neither agree nor disagree Agree Strongly agree

○ Prefer not to answer

g. The toolkit covers all the important HPV vaccine topics.
Strongly disagree Disagree Neither agree nor disagree Agree Strongly agree

○ Prefer not to answer

h. The posters are ok to hang in dental office waiting/patient rooms.
Strongly disagree Disagree Neither agree nor disagree Agree Strongly agree

○ Prefer not to answer

i. Dental patients would use these materials to learn more about the HPV vaccine.
Strongly disagree Disagree Neither agree nor disagree Agree Strongly agree

○ Prefer not to answer

j. If my dental provider shared these materials with me, I would feel comfortable talking about the HPV vaccine with them.
Strongly disagree Disagree Neither agree nor disagree Agree Strongly agree

○ Prefer not to answer

k. After reviewing these materials, my thoughts about the HPV vaccine have changed.
Strongly disagree Disagree Neither agree nor disagree Agree Strongly agree

○ Prefer not to answer

l. Additional comments about items you agreed/disagreed with about the toolkit's content:
____

HPV Education Material Review by Email Survey: Dental Providers

VIDEO:

Overall

a. Overall, the video was interesting.
Strongly disagree Disagree Neither agree nor disagree Agree Strongly agree
○ Prefer not to answer

b. I understood what the video was trying to teach me.
Strongly disagree Disagree Neither agree nor disagree Agree Strongly agree
○ Prefer not to answer

c. Nothing in the video was offensive to me.
Strongly disagree Disagree Neither agree nor disagree Agree Strongly agree
○ Prefer not to answer

d. Additional comments about items you agreed/disagreed with about the video overall:
____

Audio

e. The speaker was easy to hear.
Strongly disagree Disagree Neither agree nor disagree Agree Strongly agree
○ Prefer not to answer

f. The video used words I could understand.
Strongly disagree Disagree Neither agree nor disagree Agree Strongly agree
○ Prefer not to answer

g. Additional comments about items you agreed/disagreed with about the video audio:
____

Visual

h. Pictures, titles, and/or graphics were helpful.
Strongly disagree Disagree Neither agree nor disagree Agree Strongly agree
○ Prefer not to answer

i. Additional comments about items you agreed/disagreed with about the video visuals:
____

Content

j. The video included information from a trusted source.
Strongly disagree Disagree Neither agree nor disagree Agree Strongly agree

○ Prefer not to answer

k. The video addresses common concerns about the HPV vaccine.
Strongly disagree Disagree Neither agree nor disagree Agree Strongly agree

○ Prefer not to answer

l. After watching the video, I think it is important for people 9-45 years old who live in Ohio to get the HPV vaccine.
Strongly disagree Disagree Neither agree nor disagree Agree Strongly agree

○ Prefer not to answer

m. It was helpful to hear about how dental providers should talk with patients about the HPV vaccine.
Strongly disagree Disagree Neither agree nor disagree Agree Strongly agree

○ Prefer not to answer

n. Additional comments about items you agreed/disagreed with about the video contents:
____

Paper Materials:

Overall Appearance

a. The colors, font, and/or pictures are appropriate.
Strongly disagree Disagree Neither agree nor disagree Agree Strongly agree

○ Prefer not to answer

b. None of the materials offend me.
Strongly disagree Disagree Neither agree nor disagree Agree Strongly agree

○ Prefer not to answer

c. The way the text is formatted makes it easy to read.
Strongly disagree Disagree Neither agree nor disagree Agree Strongly agree

○ Prefer not to answer

d. Additional comments about items you agreed/disagreed with about the toolkit’s overall appearance: ____

Content

e. The messages in the materials are useful.
Strongly disagree Disagree Neither agree nor disagree Agree Strongly agree

○ Prefer not to answer

f. The printed materials address common concerns about the HPV vaccine.
Strongly disagree Disagree Neither agree nor disagree Agree Strongly agree

○ Prefer not to answer

g. The toolkit covers all the important HPV vaccine topics.
Strongly disagree Disagree Neither agree nor disagree Agree Strongly agree

○ Prefer not to answer

h. The posters are ok to hang in dental office waiting/patient rooms.
Strongly disagree Disagree Neither agree nor disagree Agree Strongly agree

○ Prefer not to answer

i. The dental provider communication tips will be helpful when talking to patients.
Strongly disagree Disagree Neither agree nor disagree Agree Strongly agree

○ Prefer not to answer

j. Dental providers and/or patients would use these materials to learn more about the HPV vaccine.
Strongly disagree Disagree Neither agree nor disagree Agree Strongly agree

○ Prefer not to answer

k. After reviewing these materials, my thoughts about the HPV vaccine have changed.
Strongly disagree Disagree Neither agree nor disagree Agree Strongly agree

○ Prefer not to answer

l. Additional comments about items you agreed/disagreed with about the toolkit's content:
____
